# Investigating the structural and functional consequences of germline single nucleotide polymorphisms located in the genes of the alternative lengthening of telomere (ALT) pathway

**DOI:** 10.1016/j.heliyon.2024.e33110

**Published:** 2024-06-18

**Authors:** Nurun Nahar Nila, Zimam Mahmud, Anik Paul, Taibur Rahman, Md. Zakir Hossain Howlader, Md. Ismail Hosen

**Affiliations:** Department of Biochemistry and Molecular Biology, University of Dhaka, Dhaka-1000, Bangladesh

**Keywords:** ALT, nsSNPs, SMARCAL1, DAXX, ATRX

## Abstract

**Background:**

The Alternative Lengthening of Telomeres (ALT) pathway represents a non-canonical mechanism of telomere maintenance that operates independently of the conventional telomerase activity. The three biologically significant proteins, designated as SMARCAL1 (SWI/SNF-related matrix-associated actin-dependent regulator of chromatin subfamily A-like protein 1), DAXX (Death domain-associated protein 6) and ATRX (alpha-thalassemia/mental retardation, X-linked) are associated with ALT in certain cancer types. The purpose of this study was to identify the most high-risk nsSNPs (non-synonymous Single Nucleotide Polymorphisms) within these three genes and assess their impacts on the structure and function of the proteins they encode.

**Methods:**

The reported genetic polymorphisms of *SMARCAL1, DAXX* and *ATRX* genes were retrieved from the Ensembl database. Later, various computational tools like PROVEAN, PolyPhen2, SNPs and GO, SNAP2, Predict-SNP, Panther and PMut were used to predict the most deleterious nsSNPs. MutPred was used to understand the underlying molecular reasons of those nsSNPs being deleterious, followed by prediction of Post Translational Modification Sites (PTMs) using ModPred. I-Mutant and MUpro were used to predict the effect of SNP on energy stability. Later, 3D clustering analysis was done using Mutation 3D server. Moreover, ConSurf was utilized to identify the conservation scores of wild-type amino acids. Additionally, the NCBI conserved domain search tool was employed to pinpoint conserved domains within these three proteins. Project-Hope helped for biophysical validation, followed by prediction of these genes’ interaction and function by using GeneMANIA.

**Result:**

Analysis on SMARCAL1 protein revealed that among 665 nsSNPs, four were identified as the most deleterious: L578S, T581S, P582A, and P582S. Similarly, within the DAXX protein, among a pool of 480 nsSNPs, P284S, R230C, and R230S were found out to be the most deleterious variants. In case of ATRX protein, V178D, R246C, and V277G, from the total of 1009 nsSNPs, were predicted to be the most deleterious. All these nsSNPs were found to occur at residue positions that are 100 % conserved within protein domains and were predicted to be most damaging from both structural and functional perspectives and highly destabilizing to their corresponding proteins.

**Conclusion:**

Computational investigation on the 3 proteins-SMARCAL1, DAXX and ATRX through different bioinformatics analysis tools concludes that the identified high risk nsSNPs of these proteins are pathogenic SNPs. These variants potentially exert functional and structural influences, thus making them valuable candidates for future genetic studies.

## 1 Introduction

The term “ALT” stands for Alternative Lengthening of Telomeres which has become one of the most important topics of the oncogenic research now a days. The reason for this is that 10–15 % of cancer subsets counteract telomere attrition during DNA replication by using a homologous recombination-based pathway called the ALT pathway rather than upregulating telomerase activity [1,2]. One of the most important steps in cancer development is the acquisition of replicative immortality, which requires both evading cell cycle checkpoints and lengthening of telomeres, regions that guard the ends of chromosomes during replication. It has recently been demonstrated that this telomerase-independent ALT mechanism extends telomeres by taking advantage of DNA repair machinery in a unique way that may provide a number of druggable targets. In various ALT + malignancies, where the average survival is inferior to that of non-ALT counterparts and the cancers display predominant resistance to conventional chemotherapeutic treatments, recognizing these targets and subsequently developing or repurposing therapies for them may be crucial for enhancing the prognosis [3].

It has been shown that ALT is frequently used by some tumor types to maintain telomere length. Particularly, mesenchymal tissue malignancies, such as those of the bone (62 %), soft tissues (32 %), neuroendocrine systems (40 %), peripheral nervous system (PNS; 23 %), and central nervous system (CNS; 15 %), predominantly employ the ALT pathway. Notably, ALT mechanism has been identified as active in a limited proportion of epithelial malignancies [4].

One of the distinguishing characteristics of ALT + cells is their telomere clustering and localization to promyelocytic leukemia (PML) bodies and this formation is termed ALT-associated PML bodies (APBs) [5]. APBs development has been found to be connected to DNA damage or replication stress at the telomere site. Additionally, APB development is accelerated by the loss of proteins such SMARCAL1, FANCD2, and FANCM that suppress replication stress or DNA damage [2,6]. This implies that the SMARCAL1 protein might act as an inhibitor of ALT.

Recent advances in cancer genome sequencing have revealed somatic mutations in ATRX and DAXX, which seem to be prevalent and unique to ALT-positive malignancies. A separate investigation revealed a correlation between ALT activity and inactivating mutations in ATRX, DAXX, and neomorphic gain-of-function H3.3 missense mutations. These mutations, which broadly impact histone methylation, were identified in 44 % of pediatric glioblastomas. Therefore, biallelic loss of function mutations of the histone chaperone DAXX and the chromatin remodeler ATRX, which are both significantly associated with ALT, may be candidates for the putative ALT suppressor [7]. The replication-independent placement of histone H3.3 at telomeres and pericentromeric chromatin is accomplished by the multifunctional chromatin remodeling histone chaperone complex that consists of ATRX and DAXX [8]. Non-functional ATRX/DAXX complex cannot assemble histone H3.3 with the chromatin and thus shows pleiotropic effects on chromatin compaction, sister chromatid cohesion, and transcription [[8], [9], [10]]. The findings on occurrence of interaction between ATRX and DNA methyltransferase 1 (DNMT1) has led to this hypothesis that ATRX deficiency is linked to the alteration of DNA methylation at sub-telomeric region [11].

Therefore, as the somatic mutations of these *SMARCAL1, DAXX* and *ATRX* genes are associated with the development of ALT mechanism, in this study we aimed at identifying the pathogenic nsSNPs (non-synonymous Single Nucleotide Polymorphism) in the 3 ALT associated genes and investigating their effect on the structure and function of their respective proteins. These findings might help in identifying the most probable candidate nsSNPs that are playing central role in the development and pathogenesis of ALT pathway. These findings will also assist in investigating on whether these nsSNPs have effects on other pathways that might open a door of simplifying the complexity of ALT pathway. Additionally, these nsSNPs can be used as diagnostic markers and might be helpful to select druggable targets which will have influence on development and repurposing of cancer therapies. This is the first computational *in silico* analysis of the whole coding regions of these 3 genes to prioritize nsSNPs for further genetic mapping studies. However, utilization of *in silico* analysis softwares speeds up identifying the deleterious nsSNPs with no cost, and also assists in future genetic studies [12].

## 2 Materials and methods

### 2.1 Data mining

The genetic variation data for the *SMARCAL1*, *DAXX* and *ATRX* genes and their respective proteins, named SMARCAL1, DAXX and ATRX were retrieved from the Ensembl database (https://asia.ensembl.org/index.html). Transcripts ENST00000357276, ENST00000266000, and ENST00000373344 are canonical transcripts for *SMARCAL1, DAXX* and *ATRX* genes respectively. From there, Protein Information Variant Table and Genetic Variant Table of these transcripts were downloaded, using “Missense Variants” and “SNP” filters. For SMARCAL1, DAXX and ATRX genes, 665, 480 and 1009 nsSNPs were retrieved, respectively. The corresponding canonical reference protein sequences of these 3 canonical transcripts were downloaded in FASTA format from the Uniprot database in which Q9NZC9, Q9UER7 and P46100 were Uniprot accession ID of SMARCAL1, DAXX and ATRX proteins, respectively.

### 2.2 Functional analysis

**Fig. 1:**
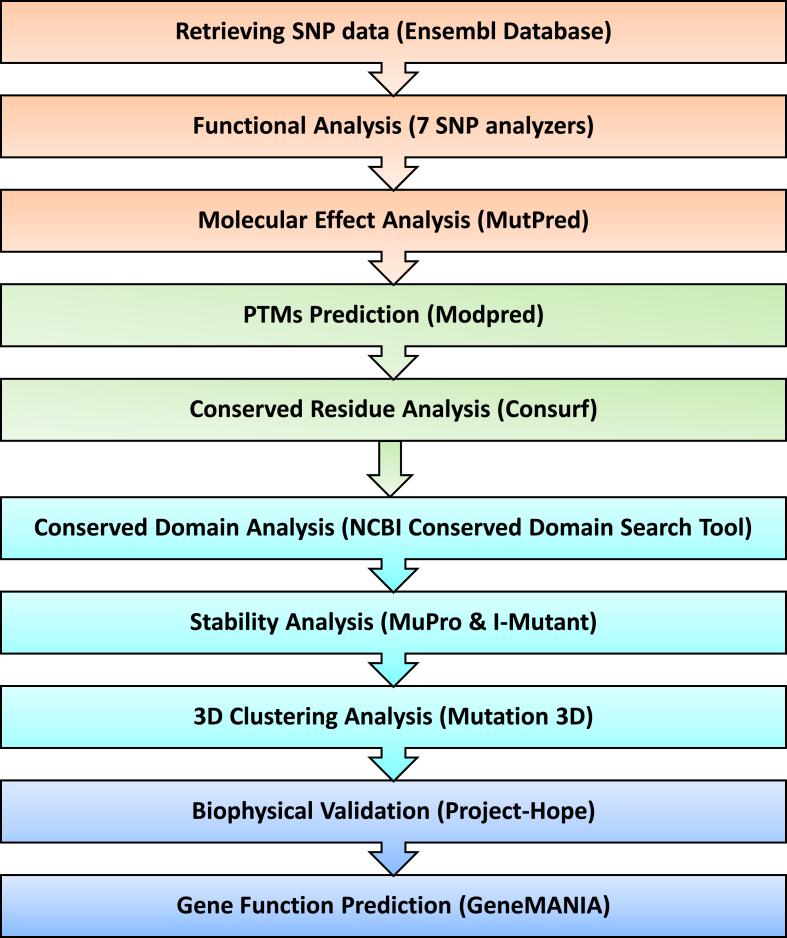
Methodology of *in silico* SNP analysis of SMARCAL1, DAXX and ATRX proteins.

In order to perform functional analysis of all the nsSNPs of these 3 target genes, 7 *in silico* SNP analyzer tools were used in batch mode: *PROVEAN, PolyPhen-2, SNPs&GO, SNAP2, PredictSNP, Panther, and PMut* (see Fig. 1). It is worth noting that *PROVEAN (Protein Variation Effect Analyzer)* can predict the impact of protein sequence variations on the biological functions of the protein. The variants are predicted as deleterious if the final score is < −2.5 and neutral if the score is > −2.5 [13,14] (http://provean.jcvi.org/protein_batch_submit.php?species=human).

*PolyPhen-2(Polymorphism Phenotyping)* is an online tool that predicts the impact of an amino acid substitution on the structure and function of the protein. Predictions are benign, possibly damaging or probably damaging. Prediction values closer to zero are regarded to be benign, whereas values closer to one are considered to likely be harmful [[15], [16], [17]] (http://genetics.bwh.harvard.edu/pph2/bgi.shtml).

*SNPs&GO* (Single nucleotide polymorphism database and gene ontology) differentiates between disease related and neutral SNPs. The output gives Prediction (either disease or neutral), Prediction Probability, and RI (probability of disease related class). The variance is disease-associated if the probability is greater than 0.5 [18,19] (https://snps.biofold.org/snps-and-go/snps-and-go.html).

*SNAP2* is a trained functional analysis tool that can distinguish between SNPs with effects and those that have no impact. It has 2 expectations: effect (positive score) or neutral (negative score). This tool also reduce runtime and enables cross-genome comparisons [20] (https://rostlab.org/services/snap2web/).

*PredictSNP* is used to forecast the impact of SNPs on the function of protein. This consensus classifier makes the nine top prediction tools to be accessed at one platform. These are: SIFT, PolyPhen-1, PolyPhen-2, MAPP, PhD-SNP, SNAP, PANTHER, PredictSNP, and nsSNPAnalyzer. By utilizing their observed accuracy values, PredictSNP provides the confidence scores produced by each instrument and a consensus prediction as percentages [21] (https://loschmidt.chemi.muni.cz/predictsnp1/).

*Panther (Protein Analysis Through Evolutionary Relationships)* uses position-specific evolutionary preservation to calculate the chance that a nsSNP will have a functional impact on the protein [22] (http://www.pantherdb.org/tools/csnpScoreForm.jsp).

*PMut* uses neural networks (NNs) trained with a vast library of neutral mutations and pathogenic mutations of mutational hot areas for quick and accurate prediction [23] P-Mut server is available at this website (http://mmb.irbbarcelona.org/PMut/).

The nsSNPs which were predicted as deleterious by all the 7 SNP analyzers, were considered as high-risk nsSNPs and further investigations were conducted for these high-risk nsSNPs.

### 2.3 Identifying molecular effects of the deleterious nsSNPs on their respective proteins

MutPred predicted the molecular basis for the deleterious effects of high-risk nsSNP's in the biological system. This includes the gain or loss of 14 distinct structural and functional properties, such as catalytic residues, Post Translational Modification Sites (PTMs), relative solvent accessibility, and structural features like helix, metal binding site, allosteric site, along with functional properties like DNA binding [24] (http://mutpred1.mutdb.org/).

### 2.4 Prediction of post translational modification site

To further explore the role of wild-type residues in their respective protein structure, cellular localization, function, and half-life within the cell, the ModPred tool was employed to predict Post Translational Modification Sites along with their modifications. It provides the name of the modifications, scores and confidence level of the prediction. Only those predictions of medium and high degree scores were considered significant. It is a useful tool in guiding biological experiments and data interpretation [25] (http://www.modpred.org/).

### 2.5 Stability analysis

To measure alteration of protein stability upon these high-risk nsSNPs, I-Mutant 3.0. and MUpro servers were used. *I-Mutant3.0* (http://gpcr2.biocomp.unibo.it/cgi/predictors/I-Mutant3.0/I-Mutant3.0.cgi). and MUpro (http://mupro.proteomics.ics.uci.edu/) calculate free energy changes to predict whether the single point mutation stabilizes or destabilizes the protein structure or not [26].

A predicted score of <0 indicates a decrease in protein stability due to the SNP, while a score of >0 suggests an increase in protein stability. Additionally, the tool provides a confidence score ranging from −1 to 1, indicating the level of confidence in the prediction [27].

### 2.6 Three-dimensional (3D) clustering analysis

The nsSNPs which decrease the stability of altered proteins were analyzed by *Mutation3D.* Mutation3D predicted clustered SNPs indicating the existence of a functional hotspot in that area. It also proposes driver genes in cancer by identifying clusters of amino acid substitutions within the tertiary protein structures [28] (http://mutation3d.org/).

### 2.7 Conservational analysis

*ConSurf* server was used for conservation analysis of the high-risk nsSNP residues of each protein. This web server offers the degree of evolutionary conservation of an amino acid in a protein of known structure in the protein data bank. It calculates conservation score from 1 to 9 for each amino acid of protein where score 1–3 are variable, 4–6 are average conserved and 7–9 are highly conserved residues [29] (https://consurf.tau.ac.il/). Furthermore, conserved domain analysis was performed by using *NCBI Conserved Domain Search tool* which annotates proteins with the location of conserved domain footprints. Therefore, this tool allows for a clear visualization of the locations of each nsSNP within various conserved domains and motifs of protein structures [30] (https://www.ncbi.nlm.nih.gov/Structure/cdd/wrpsb.cgi).

### 2.8 Biophysical validation

The nsSNPs which were predicted to be clustered SNPs by *Mutation3D*, having conservation score of 9 (ConSurf), DDG value of ≤ - 0.5 (I-Mutant and MUpro) and SNAP2 score of ≥40, were subjected to be analyzed by *Project HOPE* (Table 6)*.* Project Hope tool analyzes and illustrates the structural and functional effects of point mutations. It validates all of the previous analysis results [31] (https://www3.cmbi.umcn.nl/hope/method/). These nsSNPs were considered as “The Most Deleterious nsSNPs”.

### 2.9 Network analysis and function prediction

Each of the 3 genes were analyzed in *GeneMANIA*, separately. This webserver helps in generating hypothesis about gene function by analyzing gene lists and prioritizing genes for functional assays. GeneMANIA finds functionally similar genes to the query gene list, using a wealth of genomics and proteomics data. It also provides gene interaction networks [32,33] (http://genemania.org/) .

## 3 Results

### 3.1 Prediction of deleterious nsSNPs by functional analysis

**Fig. 2:**
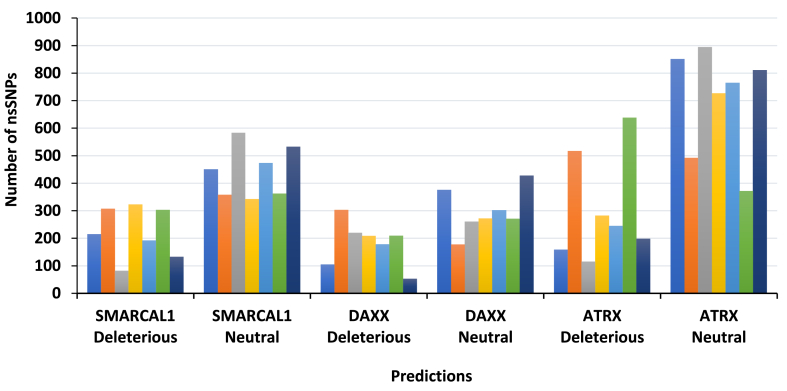
Predictions of the effects of nsSNPs of SMARCAL1, DAXX and ATRX proteins by SNP Analyzers.

The PROVEAN, PolyPhen-2, SNPs&GO, SNAP2, PredictSNP, Panther, and PMut predicted 215, 307, 82, 323, 192, 303, and 132 nsSNPs in the *SMARCAL1* gene, respectively, as deleterious or probably damaging (Fig. 2). Rest of the nsSNPs were predicted to be Neutral/Benign. In case of DAXX, out of 480 nsSNPs, PROVEAN, PolyPhen-2, SNPs&GO, SNAP2, PredictSNP, Panther and PMut predicted 104, 303, 220, 208, 178, 209, and 52 nsSNPs to be deleterious, accordingly (Fig. 2). Whereas for ATRX protein, the number of nsSNPs predicted to be deleterious by PROVEAN, PolyPhen-2, SNPs&GO, SNAP2, PredictSNP, Panther and P-Mut tools were 158, 517, 114, 282, 244, 638, and 198, respectively, as indicated in Fig. 2.

The number of high-risk nsSNPs of SMARCAL1, DAXX and ATRX were 36, 39 and 15 respectively. The predictions and scores of SNP analyzing tools for the high-risk nsSNPs of SMARCAL1, DAXX and ATRX proteins are given in the supplementary files (Table 1 to Table 6). In addition, the predictions of functional analysis of the most deleterious nsSNPs of these 3 genes are shown in Table 1, Table 2.

**Table 1:** Predictions of PROVEAN, PolyPhen-2 & SNPs&GO for the most deleterious nsSNPs of SMARCAL1, DAXX and ATRX proteins.

Gene	AA Variant	rs ID	PROVEAN		PolyPhen-2		SNPs&GO	
			Prediction	Score	Prediction	Probability	Prediction	Probability
**SMARCAL 1**	L578S	rs1445488994	Deleterious	−5.96	probably damaging	1	Disease	0.806
	T581S	rs914843328	Deleterious	−3.97	probably damaging	1	Disease	0.654
	P582A	rs755247940	Deleterious	−7.93	probably damaging	0.999	Disease	0.74
	P582S	rs755247940	Deleterious	−7.93	probably damaging	1	Disease	0.833
**DAXX**	P284S	rs1554282793	Deleterious	−7.69	probably damaging	1	Disease	0.912
	R230C	rs771876073	Deleterious	−6.42	probably damaging	1	Disease	0.828
	R230S	rs771876073	Deleterious	−4.1	probably damaging	1	Disease	0.803
**ATRX**	R246C	rs122445105	Deleterious	−4.46	probably damaging	1	Disease	0.921
	V178D	rs1060499759	Deleterious	−3.78	probably damaging	1	Disease	0.852
	V277G	rs797044793	Deleterious	−3.63	probably damaging	1	Disease	0.606

**Table 2:** Predictions of SNAP2, PredictSNP, Panther, & PMut for the most deleterious nsSNPs of SMARCAL1, DAXX and ATRX proteins.

Gene	AA Variant	SNAP2		PredictSNP		Panther	PMut	
		Prediction	Score	Prediction	Accuracy	Prediction	Disease Prediction	Score
**SMARCAL1**	L578S	effect	76	DELETERIOUS	0.86908365	probably damaging	TRUE	0.8783
	T581S	effect	79	DELETERIOUS	0.7556615	probably damaging	TRUE	0.8667
	P582A	effect	58	DELETERIOUS	0.7556615	probably damaging	TRUE	0.8667
	P582S	effect	64	DELETERIOUS	0.7556615	probably damaging	TRUE	0.8667
**DAXX**	P284S	effect	71	DELETERIOUS	0.7556615	probably damaging	TRUE	0.7993
	R230C	effect	63	DELETERIOUS	0.7556615	possibly damaging	TRUE	0.6739
	R230S	effect	70	DELETERIOUS	0.7556615	possibly damaging	TRUE	0.6739
**ATRX**	R246C	effect	58	DELETERIOUS	0.869084	probably damaging	TRUE	0.8704
	V178D	effect	40	DELETERIOUS	0.718713	probably damaging	TRUE	0.8581
	V277G	effect	45	DELETERIOUS	0.755662	probably damaging	TRUE	0.8701

### 3.2 Identification of molecular effects of the deleterious nsSNPs on mutated proteins

MutPred predicted molecular effects of the most deleterious nsSNPs of SMARCAL1, DAXX and ATRX proteins which are shown in Table 3. Additionally, the molecular effects of the high-risk nsSNPs of these 3 proteins are given in Supplementary Table 7.

**Table 3:** Molecular Effects of the most deleterious nsSNPs on their respective proteins, SMARCAL1, DAXX and ATRX.

Gene	AA Variant	Molecular mechanisms with P-values ≤ 0.05
**SMARCAL1**	L578S	Altered Ordered interface, Gain of Catalytic site at S579, Altered DNA binding, Loss of Allosteric site at T581, Altered Disordered interface, Altered Metal binding, Altered Stability
	T581S	Gain of Catalytic site at S579, Altered DNA binding, Loss of Allosteric site at T581, Altered Metal binding
	P582A	Altered Ordered interface, Gain of Helix, Loss of Catalytic site at S579, Altered DNA binding, Loss of Allosteric site at T581, Altered Metal binding
	P582S	Altered Ordered interface, Gain of Catalytic site at S579, Altered DNA binding, Altered Metal binding, Gain of Allosteric site at T581
**DAXX**	P284S	Altered Disordered interface, Gain of Relative solvent accessibility, Gain of Phosphorylation at Y286, Altered DNA binding, Gain of Proteolytic cleavage at D285, Loss of Sulfation at Y286
	R230C	–
	R230S	–
**ATRX**	R246C	Altered Metal binding, Altered Transmembrane protein, Altered DNA binding, Gain of Amidation at L248
	V178D	Gain of Catalytic site at C174, Altered Transmembrane protein
	V277G	Altered Stability

### 3.3 Prediction of post translational modification (PTM) sites

The resultant PTMs at residues of high-risk nsSNPs of these 3 proteins are shown in Supplementary Table 8. Moreover, predicted PTMs at the most deleterious nsSNP residues are given in Table 4.

**Table 4:** Prediction of PTMs at the most deleterious nsSNP residue positions of DAXX, SMARCAL1 and ATRX protein.

SMARCAL1		DAXX		ATRX	
**Residues**	PTMs	**Residues**	PTMs	**Residues**	PTMs
**L578**	Proteolytic cleavage	**R230**	ADP-ribosylation	**R246**	Proteolytic cleavage
**T581**	Phosphorylation	**P284**	Proteolytic cleavage	V178	–
**T581**	O-linked glycosylation	–	–	V277	–

### 3.4 Protein stability alteration analysis

Protein stability changes resulting from high-risk nsSNPs were pedicted by I-Mutant and MUpro. The corresponding information for SMARCAL1, DAXX, and ATRX proteins is presented in Supplementary Table 9, Table 10, and Table 11, respectively. In addition, protein stability changes upon the most deleterious nsSNPs are given in Table 5.

**Table 5:** Prediction of Protein Stability of SMARCAL1, DAXX and ATRX proteins by MuPro, and I-Mutant and Conservation status by Consurf. [e = exposed, f = functional, b = buried, s = structural residue; 9 is the highest conservation score].

Gene	AA Variant	MuPro		I-Mutant		Conservation Status
		Prediction	DDG Value (Kcal/mol)	Prediction	DDG Value (Kcal/mol)	
**SMARCAL1**	L578S	Decrease	−1.58	Decrease	−2.42	9 (b,s)
	T581S	Decrease	−0.889	Decrease	−0.77	9 (b,s)
	P582A	Decrease	−1.08	Decrease	−1.6	9 (e,f)
	P582S	Decrease	−0.817	Decrease	−1.88	9 (e,f)
**DAXX**	R230C	Decrease	−1.09	Decrease	−0.76	9 (e,f)
	R230S	Decrease	−1.29	Decrease	−1	9 (e,f)
	P284S	Decrease	−1.09	Decrease	−1.98	9 (e,f)
**ATRX**	V178D	Decrease	−0.767	Decrease	−1.07	9 (b,s)
	R246C	Decrease	−0.637	Decrease	−0.97	9 (e,f)
	V277G	Decrease	−2.665	Decrease	−2.51	9 (b,s)

**Table 6:** Project-Hope Illustrations for the most deleterious nsSNPs.

Gene	AA Variant	Hydrophobicity	Hydrophilicity	Size	Charge	Others in Wild Type	Others in Variant
SMARCAL1	L578S	Decrease	Increase	Smaller	–	Branched, Hydrophobic interaction	-OH group, may form, H-bond, empty space,
	T581S	Decrease	Increase	Smaller	–	Buried, Functional role	Empty space, altered interaction
	P582A	Decrease	–	Smaller	–	Proline Ring, rigidity, special backbone,	Less branched, Disturbed function
	P582S	Decrease	Increase	Smaller	–	Proline Ring, rigidity, special backbone	Less branch, –OH group, Disturbed function
DAXX	R230C	Increase	Decrease	Smaller	Po > Nu	Exposed, functional, ionic interaction	-SH group, may form di-sulfide bond, loss of multimeric interaction
	R230S	Increase	Decrease	Smaller	Po > Nu	Exposed, functional, ionic interaction	-OH group, may form, H-bond, disturbed ionic interaction,
	P284S	Decrease	Increase	Smaller		Histone H3.3 and H4 interface, Proline Ring, rigidity	Empty space, decreased Hydrophobic Interaction
ATRX	V178D	Decrease	Increase	Larger	Nu > Ne	Hydrophobic interaction, buried,	Disturbed folding, Ionic interaction, unaccommodated,
	R246C	Increase	Decrease	Smaller	Po > Nu	Ionic interaction, salt bridge, multimer contact	-SH group, may form di-sulfide bond, loss of salt bridge,
	V277G	Decrease	–	Smaller	–	Hydrophobic interaction, buried	Flexible, loss of rigidity, empty space

Abbreviations: Po=Positive, Nu = Neutral, Ne=Negative, “>” = changed to.

### 3.5 Three-dimensional (3D) clustering analysis

**Fig. 3:**
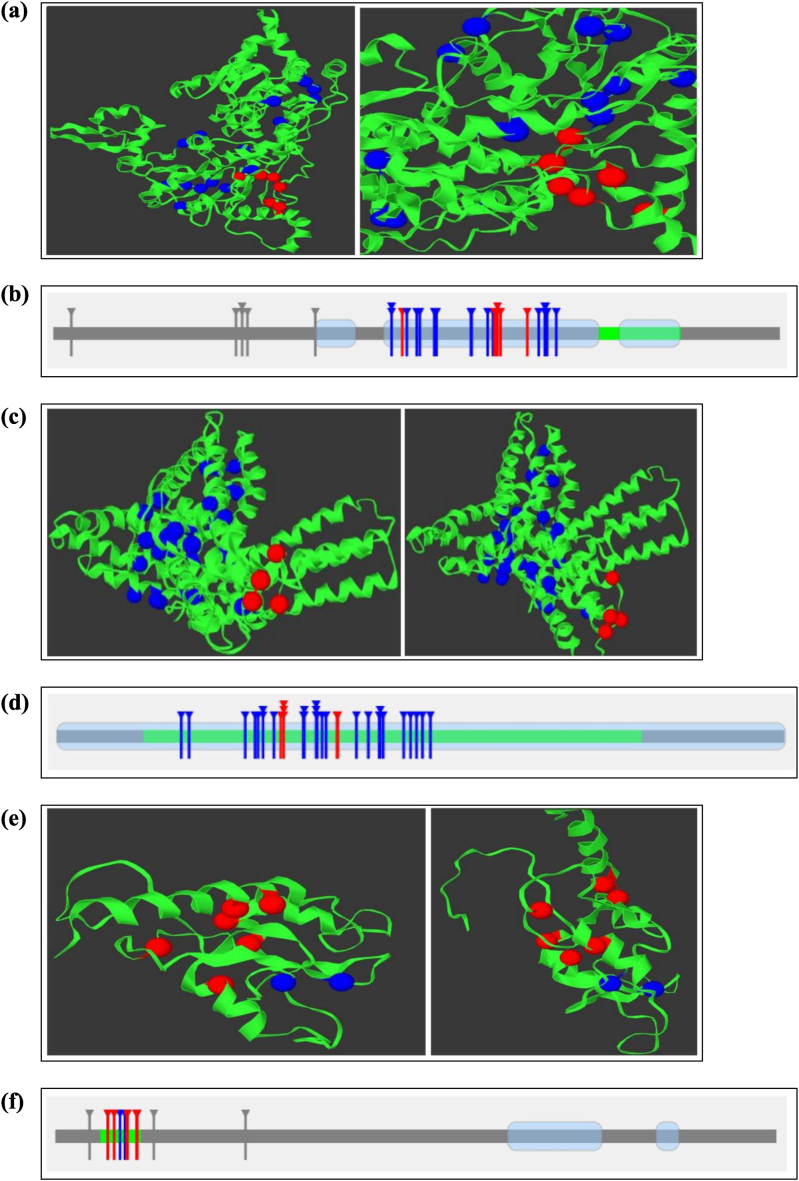
(a) Clustered nsSNPs (Red) in SMARCAL1 (from different angle). (b) Location of Clustered nsSNPs (Red), Covered (Blue) & Uncovered (Gray) nsSNPs within SMARCAL1 protein. (c) Clustered nsSNPs of DAXX Protein (Red) (from different angle). (d) Positions of Clustered (Red) & covered (Blue) nsSNPs in DAXX Protein (no uncovered nsSNP). (e) Clustered nsSNPs (Red) in ATRX protein. (from different angle). (f) Locations of Clustered (Red), covered (Blue) & uncovered (Gray) nsSNPs in ATRX protein. (For interpretation of the references to colour in this figure legend, the reader is referred to the Web version of this article.)

Mutation3D analysis identified a cluster of 7 nsSNPs within SMARCAL1, specifically the variants A457T, L578S, T581S, P582A, P582S, R586W, and G621R, among the 31 protein destabilizing nsSNPs of SMARCAL1 (Fig. 3(a) and (b)). For DAXX, the number of clustered nsSNPs were 6 (D285E, P284S, R227W, R230C, R230L, R230S) from the total of 34 protein destabilizing high-risk nsSNPs (Fig. 3(c) and (d)). By similar analysis of ATRX, out of the 11 protein stability decreasing nsSNPs, 6 nsSNPs (C200F, C243W, C280R, R246C, V178D, V277G) were predicted to be clustered nsSNPs (Fig. 3(e) and (f)).

### 3.6 Phylogenetic conservational analysis

#### 3.6.1 Conserved residue analysis

According to ConSurf prediction, 31 of the 36 nsSNP residues of SMARCAL1, 27 of the 39 nsSNP residues of DAXX and 9 of the 15 nsSNP residues of ATRX protein are 100 % conserved with conservation score 9. The conservation analysis output for the high-risk nsSNP residues for SMARCAL1, DAXX and ATRX proteins are summarized in Supplementary Table 9, 10 and 11. Furthermore, the conservational status of the most deleterious residues of these 3 proteins are shown in short in Table 5.

#### 3.6.2 Conserved domain analysis

**Fig. 4:**
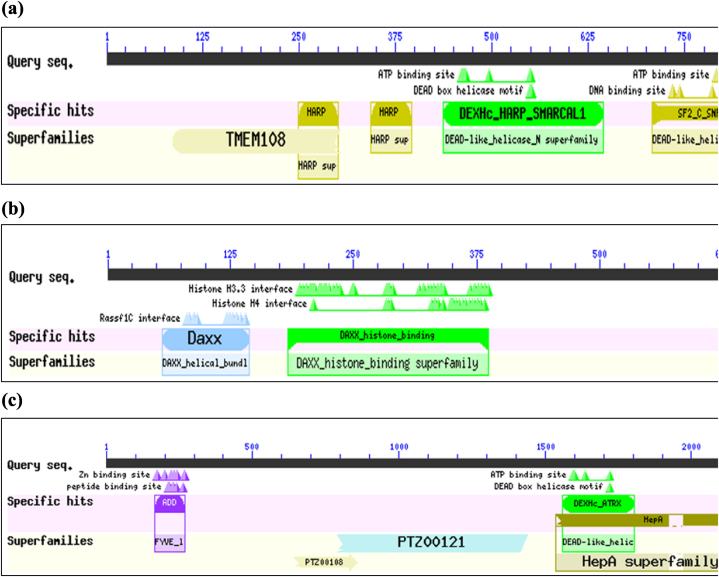
(a) NCBI Conserved Domain Search tool predicted Conserved Domains of SMARCAL1. (b) NCBI Conserved Domain Search tool predicted Conserved Domains of DAXX. (c) NCBI Conserved Domain Search tool predicted Conserved Domains of ATRX.

**Fig. 5:**
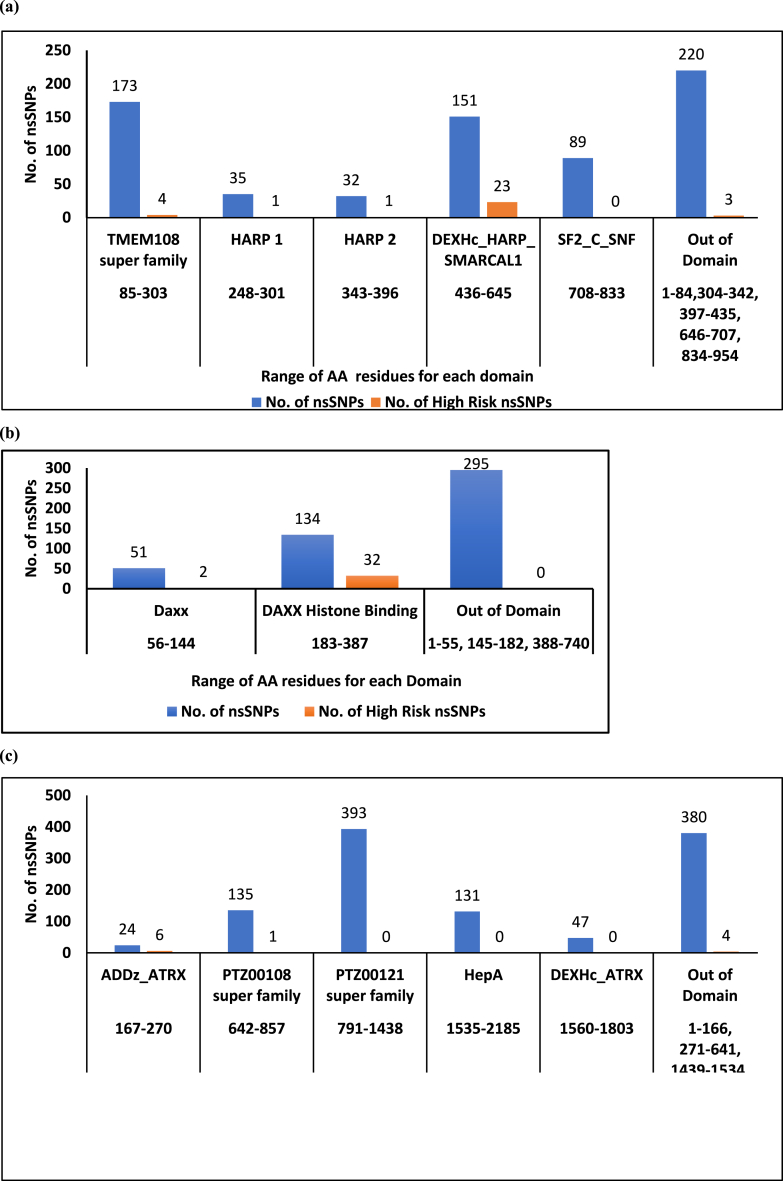
(a) Graphical representation of the comparative distribution of all the 665 nsSNPs and High-Risk nsSNPs in the conserved domains of SMARCAL1 protein. (b) Graphical representation of the comparative distribution of all the 480 nsSNPs and High-Risk nsSNPs in the conserved domains of DAXX protein. (c) Graphical representation of the comparative distribution of all the 1009 nsSNPs and High-Risk nsSNPs in the conserved domains of ATRX protein.

The results of NCBI conserved domain search tool indicated that there are 5 conserved domains of SMARCAL1 named TMEM108 Super Family Domain, HARP1, HARP2, DEXHc_HARP_SMARCAL1, and SF2_C_SNF Domain (Fig. 4(a)). In DAXX protein, 2 domains designated as Daxx Domain and DAXX Histone Binding Domain were predicted as conserved domains (Fig. 4(b)). For the ATRX protein, five domains, namely ADDz_ATRX, PTZ00108 superfamily, PTZ00121 superfamily, HepA, and DEXHc_ATRX, were identified as conserved domains (Fig. 4(c)). Furthermore, Fig. 5(a) depicts the comparative distribution of all nsSNPs from the Ensembl Database, highlighting high-risk nsSNPs inducing protein instability within conserved domains for SMARCAL1, while Fig. 5(b) and (c) show the corresponding distributions for DAXX and ATRX proteins, respectively.

### 3.7 Biophysical validation

An overview of the illustrations and biophysical validation of the most deleterious nsSNPs’ effects on their respective proteins is shown in Table 6 and Fig. 7, Fig. 8, Fig. 9, as predicted by the Project-Hope tool. The structural alterations of amino acids and their neighboring regions upon the most deleterious nsSNPs of SMARCAL1, DAXX and ATRX proteins are shown in Fig. 7, Fig. 8, Fig. 9, respectively.

### 3.8 GeneMANIA

**Fig. 6:**
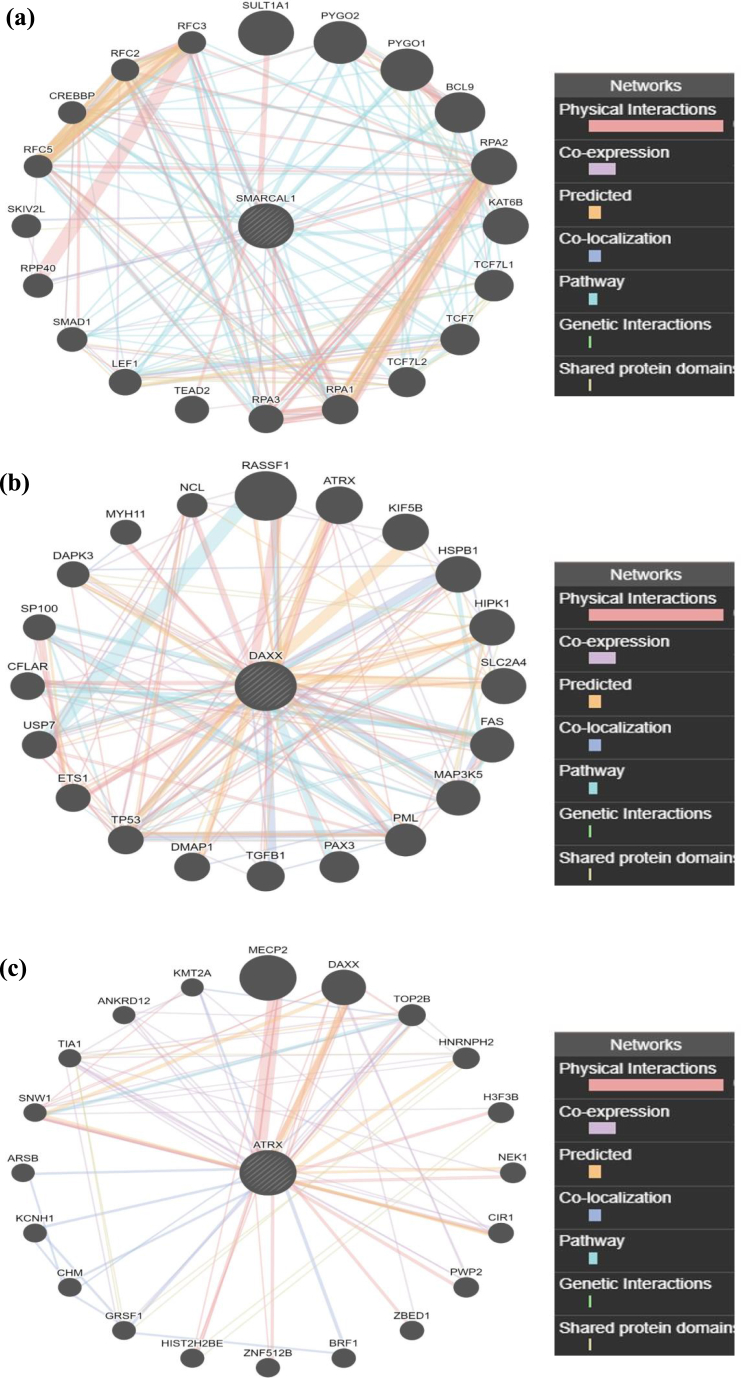
(a) GeneMANIA predicted networks of SMARCAL1. (b) GeneMANIA predicted networks of DAXX. (c) GeneMANIA predicted networks of ATRX.

Networks of gene interactions of SMARCAL1, DAXX and ATRX are shown in Fig. 6(a) and (b) and 6(c), to have a bird's eye view. GeneMANIA predicted “Pathway” and “Physical Interaction” of SMARCAL1, DAXX and ATRX are shown in Table 7. Moreover, GeneMANIA predicted functions of SMARCAL1, DAXX and ATRX are shown in Table 8, Table 9, Table 10.

**Fig. 7:**
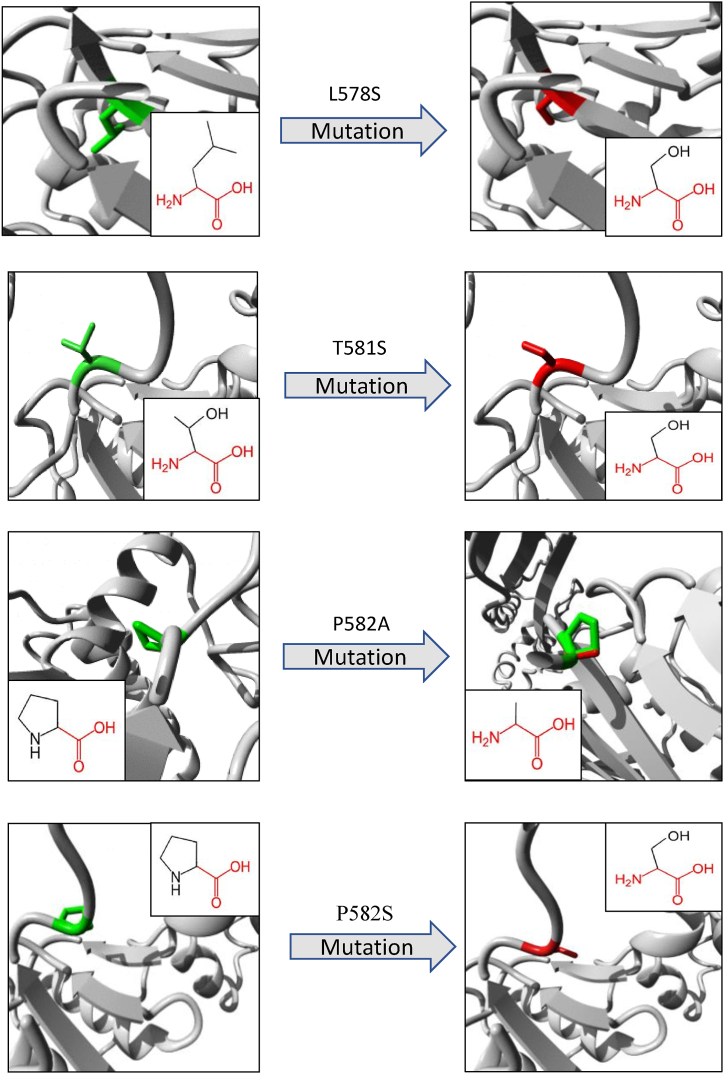
Visual representation of amino acid changes caused by SMARCAL1 SNPs from Project-HOPE. A total number of 4 mostly deleterious SNPs in the SMARCAL1 gene were selected for this analysis.

**Fig. 8:**
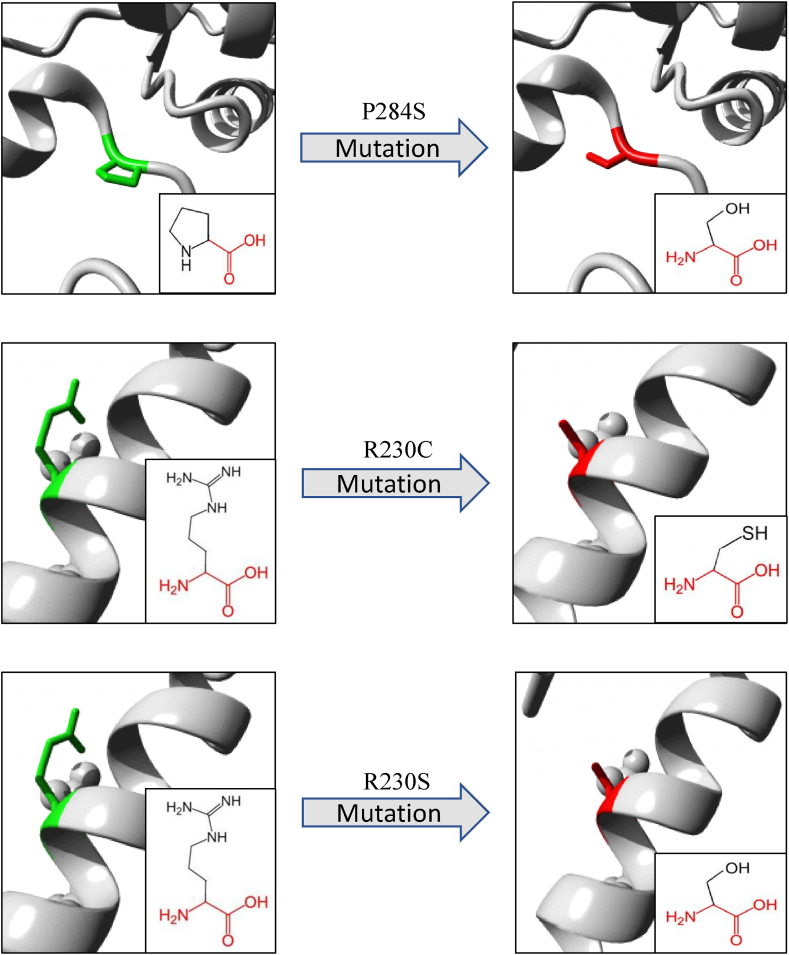
Visual representation of amino acid changes caused by DAXX SNPs from Project-HOPE. A total number of 3 mostly deleterious SNPs in the DAXX gene were selected for this analysis.

**Fig. 9:**
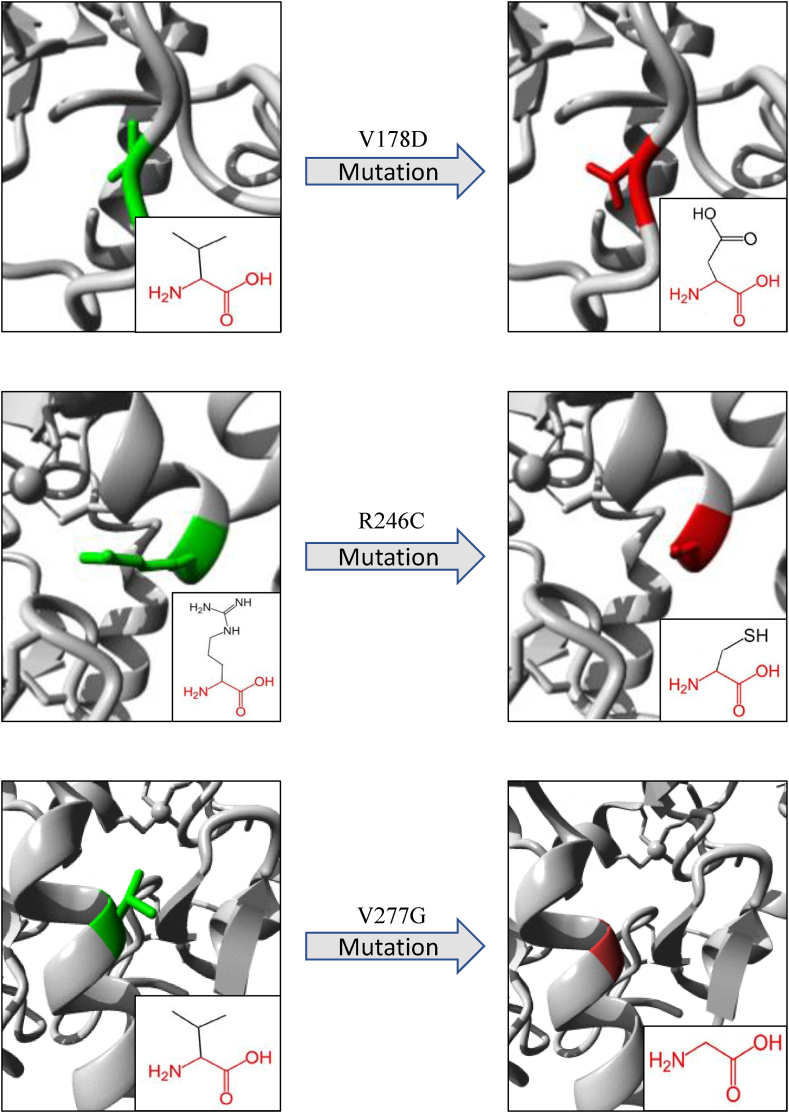
Visual representation of amino acid changes caused by ATRX SNPs from Project-HOPE. A total number of 3 mostly deleterious SNPs in the ATRX gene were selected for this analysis.

**Table 7:** GeneMANIA predicted “Pathway” and “Physical Interaction” of SMARCAL1, DAXX and ATRX. Here, *Network weight* reflects the data source relevance for predicting the function of interest.

Pathway			Physical Interactions		
Gene 1	Gene 2	Weight	Gene 1	Gene 2	Weight
PYGO2	SMARCAL1	0.0750142	SULT1A1	SMARCAL1	0.3414679
PYGO1	SMARCAL1	0.0750142	RPA2	SMARCAL1	0.1991039
BCL9	SMARCAL1	0.0731877	TEAD2	SMARCAL1	0.1709063
KAT6B	SMARCAL1	0.0630102	RPA1	SMARCAL1	0.1509552
RPA1	SMARCAL1	0.0606392	RPA2	SMARCAL1	0.0434838
RFC5	SMARCAL1	0.0605242	RPA3	SMARCAL1	0.0427389
RFC2	SMARCAL1	0.0605242	MYH11	DAXX	0.8919878
RFC3	SMARCAL1	0.0605242	ATRX	DAXX	0.7096664
RPA2	SMARCAL1	0.0580001	RASSF1	DAXX	0.5455926
RASSF1	DAXX	0.2526744	ETS1	DAXX	0.4831168
USP7	DAXX	0.2526744	PML	DAXX	0.4831168
PAX3	DAXX	0.1288288	NCL	DAXX	0.3148135
SP100	DAXX	0.0856731	MECP2	ATRX	1
FAS	DAXX	0.0730206	DAXX	ATRX	0.7096664
HSPB1	DAXX	0.0584544	H3F3B	ATRX	0.2654278
MAP3K5	DAXX	0.0572654	PWP2	ATRX	0.2359579
CFLAR	DAXX	0.039677	ZNF512B	ATRX	0.2159408
PML	DAXX	0.0371909	ZBED1	ATRX	0.2118873

**Table 8:** GeneMANIA predicted functions of SMARCAL1 gene with their FDR (False Discovery Rate) value. The less the FDR value the less possibility it has to be a false positive result/prediction.

Function	FDR	Genes in network	Genes in genome
nucleotide-excision repair, DNA gap filling	3.65591E-10	6	19
telomere maintenance via recombination	1.02933E-09	6	26
nuclear cell cycle DNA replication	1.26161E-09	6	28
DNA strand elongation involved in DNA replication	2.99069E-09	6	34
replication fork	3.24423E-09	6	36
telomere organization	5.5984E-08	6	61
anatomical structure homeostasis	1.80224E-05	6	166
protein-DNA complex assembly	0.000731067	4	70
nucleotide-excision repair, DNA damage removal	0.001093975	3	21
regulatory region DNA binding	0.004670802	5	268
canonical Wnt signaling pathway	0.009017853	4	145
chromatin binding	0.009496244	4	148
double-strand break repair via homologous recombination	0.014242644	3	55
histone acetylation	0.072585261	3	102
internal peptidyl-lysine acetylation	0.073121689	3	103

**Table 9:** GeneMANIA predicted functions of DAXX gene with their FDR (False Discovery Rate) value.

Function	FDR	Genes in network	Genes in genome
regulation of angiogenesis	0.001135056	5	131
PML body	0.001298262	4	57
regulation of endothelial cell migration	0.003337448	4	76
cellular response to oxidative stress	0.006356554	4	95
regulation of epithelial cell migration	0.006745862	4	99
DNA methylation	0.012674893	3	38
negative regulation of cell cycle	0.012674893	5	290
DNA alkylation	0.012674893	3	38
p53 binding	0.01586832	3	48
DNA methylation or demethylation	0.018534927	3	52
regulation of histone deacetylation	0.032584556	2	11
DNA damage response, signal transduction by p53	0.050976089	2	14
positive regulation of apoptotic process	0.06074056	4	249
regulation of protein deacetylation	0.06074056	2	16
response to oxygen levels	0.082983577	3	120

**Table 10:** GeneMANIA predicted functions of ATRX protein.

Function	FDR	Genes in network	Genes in genome
chromatin	0.689990329	4	213
heterochromatin	0.111269319	3	33

## 4 Discussion

In this study, we kept our focus on *in silico* investigation of the impact of genetic nsSNPs on the structural and functional behavior of SMARCAL1, DAXX and ATRX. All these 3 proteins are negatively associated with the ALT pathway. ALT pathway acts as an alternative way of telomere maintenance to overcome replicative senescence by 10–15 % of cancer diseases [1].

A combination of 7 *in silico* tools were used to predict the deleterious effects of SNPs on their corresponding proteins. These nsSNPs which are predicted to be deleterious by all these SNP analyzers hold a strong background to be highly deleterious or of high-risk for its negative impact on cellular and biological processes (Supplementary Tables 1–6).

However, from thermodynamic perspective, it is also necessary to see whether the SNPs affects the stability of the protein positively or negatively. Hence, following the predictions from I-Mutant and MUpro, five nsSNPs in the SMARCAL1 protein were excluded from further analysis. (Supplementary Table 9). In case of DAXX protein, 5 nsSNPs were predicted to be protein stability increasing SNPs and thus, these 5 SNPs were not considered for further analysis (Supplementary Table 10). Whereas for ATRX protein, the number of protein stability increasing nsSNPs were 4 (Supplementary Table 11).

To gain a deeper understanding of the significance of an amino acid residue in a protein's behavior, it is more effective and reliable approach to assess the conservation profile of that particular amino acid residue. Moreover, assessing the profound impact of a substitution on a modified protein is more meaningful and productive when considering the conservation status alongside the function of the domain where the substitution is located. For example, DEXHc_HARP_SMARCAL1, SF2_C_SNF, HARP1, HARP2, TMEM108 super family are the Domain Hits of SMARCAL1 protein (Fig. 6(a)). Herein, HARP1 and HARP2 (HepA-related protein) domains exhibit single-stranded DNA-dependent ATPase activity. In contrast, the SF2–C–SNF domain serves as the C-terminal helicase domain involved in ATP-dependent DNA unwinding. This implies that the SF2–C–SNF domain harbors both DNA and ATP binding sites.

Furthermore, NCBI conserved domain search tool predicted that all the clustered mutations are located in the DEXHc-HARP-SMARCAL1 domain, which is also implicated in ATP-dependent DNA unwinding. This domain has an ATP binding site and a Dead Box Helicase Motif. Additionally, it has been found that 23 of the 36 nsSNPs reside in this domain (Fig. 7(a)). Overall, considering both statistical and qualitative findings, it can be asserted that the DEXHc_HARP_SMARCAL1 domain appears to be the most susceptible domain. In this domain, the occurrence of any deleterious mutation might have many folds’ negative effect on the structure and functional features of SMARCAL1 protein rather than having mutation in other domains.

In case of DAXX protein, NCBI conserved domain search tool predicted 2 conserved domains in it, namely, DAXX_histone_binding Domain (Histone binding domain of the death-domain associated protein (DAXX)) and Daxx Domain (Daxx N-terminal Rassf1C-interacting domain) (Fig. 6(b)). Daxx domain contains Rassf1C interface. This interface binds to the N-terminal residues of the tumor-suppressor protein named Rassf1C (Ras-association domain family 1C). Moreover, all the clustered nsSNPs are located in histone binding domain. Within this domain, there exists both the Histone H3.3 interface and the Histone H4 interface, enabling interaction with the histone H3.3-H4 dimer. By doing this, it competes with DNA binding and the interactions involving the histone chaperone ASF1/CIA and the H3–H4 dimer. It is worth noting that the DAXX protein also functions as a histone chaperon [34].

Statistically, it has been found that 2 high-risk nsSNP residues of DAXX protein resides in Daxx-Rassf1C Interacting Domain, while 32 are in the Histone Binding Domain (Fig. 7(b)).

Overall, these quantitative as well as qualitative findings suggest that the most vulnerable and possible prolific target of cancer for disposing mutations can be the DAXX_Hsitone_Binding Domain. This domain is also a functional hotspot of DAXX protein, as predicted by Mutation 3D server. Therefore, this might result in the inability of DAXX protein to act as an effective Histone Chaperon or to keep pace in competition with ASF1/CIA chaperon [34].

Regarding ATRX protein, ADDz_ATRX, DEXHc_ATRX, HepA, PTZ00121 super family, PTZ00108 super family, are the 5 conserved domains (Fig. 6(c)). The ADDz_ATRX domain contains Zn and Peptide binding sites. This domain recognizes a specific methylated histone, which interaction is required for heterochromatin localization of the ATRX protein. DEXHc_ATRX, the DEXH-box helicase domain of ATRX is involved in ATP-dependent DNA unwinding. Thus, this domain harbors both an ATP-binding site and a DEAD box helicase motif. However, it is noteworthy that six of the High Risk nsSNPs (Fig. 7(c)) and four of the clustered nsSNPs are located in the ADDz_ATRX domain. Hence, the results suggest that the ADDz_ATRX domain is likely the most susceptible and probable target for mutations in cancer cells. This assertion is reinforced by the Mutation3D server's prediction, which identifies this domain as a functional hotspot.

In order to narrow down the selection of the most deleterious nsSNPs, specific criteria were established, including a SNAP2 score of ≥40, a DDG value < −0.5 in both I-Mutant and MUpro, and a Consurf's Conservation Score of 9. The most deleterious nsSNPs of SMARCAL1 protein were L578S, T581S, P582A and P582S. And as for DAXX protein, P284S, R230C and R230S were the most deleterious nsSNPs. In case of ATRX protein, V178D, R246C, and V277G were selected as the most deleterious nsSNPs.

Upon closer examination of the molecular impacts of these most deleterious nsSNPs from both functional and structural standpoints, it becomes evident that the L578S, T581S, P582A, and P582S nsSNPs lead to modifications in the DNA and metal binding capabilities of the SMARCAL1 protein (Table 3). Moreover, the effect on the function of the SMARCAL1 protein resulting from the changed DNA binding can be readily understood by examining GeneMANIA's prediction of its function (Table 8). GeneMANIA states that SMARCAL1 protein has transcription regulatory region sequence-specific DNA binding activity with FDR value 0.053. Consequently, the targeted outcome of transcription regulation will not be achieved. Likewise, since the function of the SMARCAL1 protein is predominantly linked to DNA processes such as telomere maintenance through semi-conservative replication/recombination, DNA-dependent DNA replication, DNA recombination, telomere organization, nucleotide-excision repair, etc. (as outlined in Table 8), the involvement of metal ions is a crucial step in these processes. Therefore, alteration in metal binding will result in unexpected impediment in SMARCAL1 protein's native functions. However, the insertion of L578S, T581S, and P582S nsSNPs results in the acquisition of a novel function, as predicted by MutPred. This newfound function involves gaining a catalytic site at S579, a residue located within the N-terminal Helicase domain of SMARCAL1. Moreover, Loss of Allosteric site at T581 is also predicted to occur due to L578S, T581S and P582A nsSNPs. It is well known that allosteric site is the regulatory site of a protein, where an effector binds and regulates the activity of that protein. Thus, loss of regulatory site indicates the loss of regulation of SMARCAL1.

It is already established that DAXX and ATRX together constitute a Multifunctional Chromatin Remodeling Histone Chaperon Complex. Our *in silico* analysis further reveals that the most deleterious nsSNPs in the DAXX protein are located within the Histone Binding domain, while the most deleterious nsSNPs in ATRX are situated in the ADDz_ATRX domain. Therefore, from these domains' function like Histone variant H3.3-H4 dimer interaction and methylated Histone recognition, it can be realized that these most deleterious nsSNPs are great threat to the Histone Variant H3.3 disposition at telomeric site. Histone H3.3 disposition is necessary because it maintains DNA in B-form, which avoids G-quadruplex formation and replication fork stalling. Therefore, disruption in DAXX and ATRX's normal functioning consequently plays significant role in Alternative Lengthening of Telomeres.

## 5 Conclusion

Investigations through different bioinformatics techniques on the reported nsSNPs of 3 ALT associated proteins concludes that, L578S, T581S, P582A and P582S nsSNPs of SMARCAL1 protein, P284S, R230C and R230S nsSNPs of DAXX protein, and V178D, R246C, and V277G nsSNPs of ATRX protein are the most high-risk nsSNPs. These nsSNPs are highly deleterious to both the structural and functional aspects of these proteins. Importantly, all of these variations are situated at highly conserved residue positions within the conserved domains of the proteins. Additionally, they are highly destabilizing to their respective proteins. Therefore, these ALT associated nsSNPs may assist in genetic studies on ALT mechanism with a special consideration of the large heterogeneity of cancers cells. Moreover, these can be strongly considered as key candidates to be used as diagnostic nsSNPs and being helpful in effective drug discovery and developing precision medicines. However, further investigations and *in vitro* experimentations are needed to explore the effects of these polymorphisms on structure and function of the protein.

## Data availability

All data generated or analyzed during this study are included in this published article.

## Funding

This research did not receive any specific grant from funding agencies in the public, commercial, or not-for-profit sectors.

## CRediT authorship contribution statement

**Nurun Nahar Nila:** Writing – review & editing, Writing – original draft, Visualization, Methodology, Investigation, Formal analysis, Data curation. **Zimam Mahmud:** Writing – review & editing, Writing – original draft, Visualization, Validation, Project administration, Methodology, Investigation, Formal analysis, Data curation, Conceptualization. **Anik Paul:** Writing – original draft, Resources, Methodology, Investigation, Formal analysis, Data curation. **Taibur Rahman:** Writing – original draft, Methodology, Investigation, Formal analysis, Data curation. **Md. Zakir Hossain Howlader:** Writing – review & editing, Supervision, Resources, Project administration, Methodology, Investigation, Formal analysis, Data curation. **Md. Ismail Hosen:** Writing – review & editing, Writing – original draft, Visualization, Validation, Supervision, Resources, Project administration, Methodology, Investigation, Formal analysis, Data curation, Conceptualization.

## Declaration of competing interest

The authors declare that they have no known competing financial interests or personal relationships that could have appeared to influence the work reported in this paper.

## References

[1] Cesare A.J., Reddel R.R. (2010). Alternative lengthening of telomeres: Models, mechanisms and implications. Nat. Rev. Genet..

[2] Zhang J.M., Zou L. (2020). Alternative lengthening of telomeres: from molecular mechanisms to therapeutic outlooks. Cell Biosci..

[3] Kent T., Gracias D., Shepherd S., Clynes D. (2020). Alternative lengthening of telomeres in pediatric cancer: mechanisms to therapies. Front. Oncol..

[4] Henson J.D., Reddel R.R. (2010). Assaying and investigating Alternative Lengthening of Telomeres activity in human cells and cancers. FEBS Lett..

[5] Henson J.D. (2005). A robust assay for alternative lengthening of telomeres in tumors shows the significance of alternative lengthening of telomeres in sarcomas and astrocytomas. Clin. Cancer Res..

[6] Lin B. (2017). 乳鼠心肌提取 HHS public access. Physiol. Behav..

[7] Jensen D.M. (2018). 肌肉作为内分泌和旁分泌器官 HHS public access. Physiol. Behav..

[8] Law M.J. (2010). ATR-X syndrome protein targets tandem repeats and influences allele-specific expression in a size-dependent manner. Cell.

[9] Goldberg A.D. (2010). Distinct factors control histone variant H3.3 localization at specific genomic regions. Cell.

[10] Ramamoorthy M., Smith S. (2015). Loss of ATRX suppresses resolution of telomere cohesion to control recombination in ALT cancer cells. Cancer Cell.

[11] Iwase S. (2012). ATRX links atypical histone methylation recognition mechanisms to human brain function Shigeki. Nat. Struct. Mol. Biol..

[12] Tenenbaum J.D. (2016). Translational bioinformatics: past, present, and future. Dev. Reprod. Biol..

[13] Choi Y. (2012).

[14] Choi Y., Sims G.E., Murphy S., Miller J.R., Chan A.P. (2012). Predicting the functional effect of amino acid substitutions and indels. PLoS One.

[15] Adzhubei I.A. (2010). A method and server for predicting damaging missense mutations. Nat. Methods.

[16] Adzhubei Ivan A., Schmidt Steffen, Peshkin Leonid, Ramensky Vasily E., Gerasimova Anna, Bork Peer, Kondrashov Alexey S., R. S S. (2010). A method and server for predicting damaging missense mutations. Nat. Methods.

[17] Human Non-Synonymous SNPs: Server and Survey - PubMed. https://pubmed.ncbi.nlm.nih.gov/12202775/. 10.1093/nar/gkf493 PMC137415 12202775

[18] Calabrese R., Capriotti E., Fariselli P., Martelli P.L., Casadio R. (2009). Functional annotations improve the predictive score of human disease-related mutations in proteins. Hum. Mutat..

[19] Turner M.R. (2016). 乳鼠心肌提取 HHS public access. Physiol. Behav..

[20] Yachdav G., Hecht M., Pasmanik-Chor M., Yeheskel A., Rost B. (2014). HeatMapViewer: interactive display of 2D data in biology. F1000Research.

[21] Bendl J. (2014). PredictSNP: robust and accurate consensus classifier for prediction of disease-related mutations. PLoS Comput. Biol..

[22] Tang H., Thomas P.D. (2016). PANTHER-PSEP: predicting disease-causing genetic variants using position-specific evolutionary preservation. Bioinformatics.

[23] Ferrer-Costa C. (2005). PMUT: a web-based tool for the annotation of pathological mutations on proteins. Bioinformatics.

[24] Li B. (2009). Automated inference of molecular mechanisms of disease from amino acid substitutions. Bioinformatics.

[25] Pejaver V. (2014). The structural and functional signatures of proteins that undergo multiple events of post-translational modification. Protein Sci..

[26] Capriotti E., Fariselli P., Casadio R. (2005). I-Mutant2.0: predicting stability changes upon mutation from the protein sequence or structure. Nucleic Acids Res..

[27] Cheng J., Randall A., Baldi P. (2006). Prediction of protein stability changes for single-site mutations using support vector machines. Proteins Struct. Funct. Genet..

[28] Meyer M.J. (2017).

[29] Ashkenazy H. (2016). ConSurf 2016: an improved methodology to estimate and visualize evolutionary conservation in macromolecules. Nucleic Acids Res..

[30] Marchler-Bauer A. (2015). CDD: NCBI's conserved domain database. Nucleic Acids Res..

[31] Venselaar H., te Beek T.A.H., Kuipers R.K.P., Hekkelman M.L., Vriend G. (2010). Protein structure analysis of mutations causing inheritable diseases. An e-Science approach with life scientist friendly interfaces. BMC Bioinf..

[32] Montojo J. (2010). GeneMANIA cytoscape plugin: fast gene function predictions on the desktop. Bioinformatics.

[33] Warde-Farley D. (2010). The GeneMANIA prediction server: biological network integration for gene prioritization and predicting gene function. Nucleic Acids Res..

[34] Long Öztekin, Nicole M., Badre D. (2008). 基因的改变NIH public access. Bone.

