# Supplementary material for: Investigating the structural and functional consequences of germline single nucleotide polymorphisms located in the genes of the alternative lengthening of telomere (ALT) pathway
Source: Heliyon. 2024 Jun 18;10(12):e33110. doi: 10.1016/j.heliyon.2024.e33110 (PMC11253002; doi:10.1016/j.heliyon.2024.e33110)
Supplement: Multimedia component 1 [file mmc1.docx]

Supplementary Table 1: Predictions of PROVEAN, PolyPhen-2 & SNPs&GO for the 36 High Risk nsSNPs of SMARCAL1 protein.

| AA Variant | rs ID | Codons | PROVEAN | | PolyPhen-2 | | SNPs&GO | |
| --- | --- | --- | --- | --- | --- | --- | --- | --- |
|  |  |  | Score | Prediction | *Probability* | *Prediction* | Prediction | Probability |
| R23C | rs753690688 | CGC > TGC | -2.71 | Deleterious | 1 | probably damaging | Disease | 0.707 |
| G239R | rs1409850348 | GGA > AGA | -2.92 | Deleterious | 1 | probably damaging | Disease | 0.618 |
| R247C | rs867970870 | CGT > TGT | -3.83 | Deleterious | 1 | probably damaging | Disease | 0.852 |
| R247P | rs1046678608 | CGT > CCT | -3.14 | Deleterious | 1 | probably damaging | Disease | 0.905 |
| Y254S | rs368040473 | TAC > TCC | -3.76 | Deleterious | 1 | probably damaging | Disease | 0.729 |
| F343S | rs1403239152 | TTC > TCC | -7.09 | Deleterious | 1 | probably damaging | Disease | 0.687 |
| G443R | rs772817540 | GGA > CGA | -7.62 | Deleterious | 1 | probably damaging | Disease | 0.931 |
| G443A | rs1158142533 | GGA > GCA | -5.66 | Deleterious | 0.877 | possibly damaging | Disease | 0.802 |
| A457T | rs778972713 | GCT > ACT | -3.97 | Deleterious | 0.999 | probably damaging | Disease | 0.742 |
| D458E | rs200566186 | GAC > GAG | -3.97 | Deleterious | 1 | probably damaging | Disease | 0.791 |
| G463E | rs1431465042 | GGG > GAG | -7.95 | Deleterious | 1 | probably damaging | Disease | 0.837 |
| K464R | rs767891287 | AAG > AGG | -2.98 | Deleterious | 1 | probably damaging | Disease | 0.763 |
| T465I | rs1178301097 | ACC > ATC | -5.96 | Deleterious | 1 | probably damaging | Disease | 0.847 |
| R476W | rs375111736 | CGG > TGG | -6.23 | Deleterious | 0.999 | probably damaging | Disease | 0.548 |
| P480L | rs758367100 | CCG > CTG | -9.93 | Deleterious | 1 | probably damaging | Disease | 0.615 |
| S487F | rs1187780569 | TCC > TTC | -5.81 | Deleterious | 0.999 | probably damaging | Disease | 0.631 |
| R499W | rs1302790588 | CGG > TGG | -5.41 | Deleterious | 0.999 | probably damaging | Disease | 0.629 |
| P502Q | rs755386504 | CCA > CAA | -7.38 | Deleterious | 0.999 | probably damaging | Disease | 0.77 |
| I547T | rs1387796277 | ATC > ACC | -4.9 | Deleterious | 1 | probably damaging | Disease | 0.752 |
| I548N | rs119473036 | ATT > AAT | -4.54 | Deleterious | 0.997 | probably damaging | Disease | 0.596 |
| L569P | rs1199493662 | CTA > CCA | -4.91 | Deleterious | 0.89 | possibly damaging | Disease | 0.588 |
| I576T | rs138819354 | ATC > ACC | -4.13 | Deleterious | 0.973 | probably damaging | Disease | 0.641 |
| L578S | rs1445488994 | TTG > TCG | -5.96 | Deleterious | 1 | probably damaging | Disease | 0.806 |
| T581S | rs914843328 | ACA > TCA | -3.97 | Deleterious | 1 | probably damaging | Disease | 0.654 |
| P582A | rs755247940 | CCA > GCA | -7.93 | Deleterious | 0.999 | probably damaging | Disease | 0.74 |
| P582S | rs755247940 | CCA > TCA | -7.93 | Deleterious | 1 | probably damaging | Disease | 0.833 |
| R586W | rs119473038 | CGG > TGG | -7.95 | Deleterious | 1 | probably damaging | Disease | 0.79 |
| C613Y | rs1187912884 | TGT > TAT | -10.9 | Deleterious | 1 | probably damaging | Disease | 0.897 |
| G621R | rs1226675532 | GGG > AGG | -7.39 | Deleterious | 0.767 | possibly damaging | Disease | 0.591 |
| L636P | rs764452445 | CTG > CCG | -5.79 | Deleterious | 0.999 | probably damaging | Disease | 0.822 |
| R644Q | rs140515579 | CGG > CAG | -3.84 | Deleterious | 1 | probably damaging | Disease | 0.802 |
| R644W | rs1313658611 | CGG > TGG | -7.68 | Deleterious | 1 | probably damaging | Disease | 0.825 |
| R645H | rs1281345070 | CGC > CAC | -4.8 | Deleterious | 1 | probably damaging | Disease | 0.795 |
| R645C | rs119473037 | CGC > TGC | -7.68 | Deleterious | 1 | probably damaging | Disease | 0.81 |
| K647E | rs1224206892 | AAG > GAG | -3.84 | Deleterious | 1 | probably damaging | Disease | 0.783 |
| R659C | rs148893764 | CGC > TGC | -7.31 | Deleterious | 1 | probably damaging | Disease | 0.786 |

Supplementary Table 2: Predictions of SNAP2, PredictSNP, Panther, & PMut for the 36 High Risk nsSNPs of SMARCAL1 protein.

| AA Variant | SNAP2 | | PredictSNP | | Panther | PMut | |
| --- | --- | --- | --- | --- | --- | --- | --- |
|  | Prediction | Score | Prediction | Accuracy | Prediction | Disease Prediction | Score |
| R23C | effect | 46 | DELETERIOUS | 0.60548272 | probably damaging | TRUE | 0.5811 |
| G239R | effect | 61 | DELETERIOUS | 0.50595948 | probably damaging | TRUE | 0.6612 |
| R247C | effect | 68 | DELETERIOUS | 0.60697259 | probably damaging | TRUE | 0.643 |
| R247P | effect | 87 | DELETERIOUS | 0.7556615 | probably damaging | TRUE | 0.5278 |
| Y254S | effect | 80 | DELETERIOUS | 0.71871275 | probably damaging | TRUE | 0.5522 |
| F343S | effect | 73 | DELETERIOUS | 0.71871275 | probably damaging | TRUE | 0.7687 |
| G443R | effect | 81 | DELETERIOUS | 0.86908365 | probably damaging | TRUE | 0.8783 |
| G443A | effect | 20 | DELETERIOUS | 0.60548272 | probably damaging | TRUE | 0.7549 |
| A457T | effect | 49 | DELETERIOUS | 0.7556615 | probably damaging | TRUE | 0.8667 |
| D458E | effect | 92 | DELETERIOUS | 0.86908365 | probably damaging | TRUE | 0.8667 |
| G463E | effect | 83 | DELETERIOUS | 0.86908365 | probably damaging | TRUE | 0.8783 |
| K464R | effect | 83 | DELETERIOUS | 0.86908365 | probably damaging | TRUE | 0.8667 |
| T465I | effect | 73 | DELETERIOUS | 0.86908365 | probably damaging | TRUE | 0.8783 |
| R476W | effect | 74 | DELETERIOUS | 0.7556615 | possibly damaging | TRUE | 0.6194 |
| P480L | effect | 27 | DELETERIOUS | 0.71871275 | probably damaging | TRUE | 0.8652 |
| S487F | effect | 65 | DELETERIOUS | 0.7556615 | probably damaging | TRUE | 0.8319 |
| R499W | effect | 29 | DELETERIOUS | 0.7556615 | probably damaging | TRUE | 0.8623 |
| P502Q | effect | 48 | DELETERIOUS | 0.7556615 | probably damaging | TRUE | 0.8652 |
| I547T | effect | 56 | DELETERIOUS | 0.86908365 | probably damaging | TRUE | 0.7216 |
| I548N | effect | 96 | DELETERIOUS | 0.86908365 | possibly damaging | TRUE | 0.8319 |
| L569P | effect | 59 | DELETERIOUS | 0.65494636 | probably damaging | TRUE | 0.5943 |
| I576T | effect | 63 | DELETERIOUS | 0.7556615 | probably damaging | TRUE | 0.8188 |
| L578S | effect | 76 | DELETERIOUS | 0.86908365 | probably damaging | TRUE | 0.8783 |
| T581S | effect | 79 | DELETERIOUS | 0.7556615 | probably damaging | TRUE | 0.8667 |
| P582A | effect | 58 | DELETERIOUS | 0.7556615 | probably damaging | TRUE | 0.8667 |
| P582S | effect | 64 | DELETERIOUS | 0.7556615 | probably damaging | TRUE | 0.8667 |
| R586W | effect | 97 | DELETERIOUS | 0.7556615 | probably damaging | TRUE | 0.8028 |
| C613Y | effect | 60 | DELETERIOUS | 0.71871275 | probably damaging | TRUE | 0.5263 |
| G621R | effect | 40 | DELETERIOUS | 0.54946365 | probably damaging | TRUE | 0.5286 |
| L636P | effect | 71 | DELETERIOUS | 0.86908365 | probably damaging | TRUE | 0.8623 |
| R644Q | effect | 72 | DELETERIOUS | 0.7556615 | probably damaging | TRUE | 0.8783 |
| R644W | effect | 88 | DELETERIOUS | 0.86908365 | probably damaging | TRUE | 0.8783 |
| R645H | effect | 82 | DELETERIOUS | 0.86908365 | probably damaging | TRUE | 0.8667 |
| R645C | effect | 96 | DELETERIOUS | 0.86908365 | probably damaging | TRUE | 0.8783 |
| K647E | effect | 77 | DELETERIOUS | 0.86908365 | probably damaging | TRUE | 0.8783 |
| R659C | effect | 64 | DELETERIOUS | 0.86908365 | probably damaging | TRUE | 0.7992 |

Supplementary Table 3: Predictions of PROVEAN, PolyPhen-2 & SNPs&GO for the 39 High Risk nsSNPs of DAXX protein.

| AA Variant | rs ID | Codon | PROVEAN | | PolyPhen-2 | | SNPs&GO | |
| --- | --- | --- | --- | --- | --- | --- | --- | --- |
|  |  |  | Prediction | Score | Prediction | Probability | Prediction | Probability |
| C74F | rs1251843066 | T**G**T > T**T**T | Deleterious | -6.95 | probably damaging | 1 | Disease | 0.893 |
| D219V | rs762108531 | G**A**C > G**T**C | Deleterious | -7.31 | probably damaging | 1 | Disease | 0.761 |
| S220C | rs764044001 | T**C**C > T**G**C | Deleterious | -4.68 | probably damaging | 1 | Disease | 0.607 |
| S102L | rs1455644209 | TCG > TTG | Deleterious | -5.21 | probably damaging | 1 | Disease | 0.886 |
| R251C | rs1477247712 | CGT > TGT | Deleterious | -7.68 | probably damaging | 1 | Disease | 0.904 |
| R328H | rs1266548613 | CGT > CAT | Deleterious | -4.81 | probably damaging | 1 | Disease | 0.945 |
| R251H | rs200273259 | CGT > CAT | Deleterious | -4.8 | probably damaging | 1 | Disease | 0.835 |
| P284S | rs1554282793 | CCT > TCT | Deleterious | -7.69 | probably damaging | 1 | Disease | 0.912 |
| E209Q | rs370297398 | GAG > CAG | Deleterious | -2.54 | probably damaging | 1 | Disease | 0.579 |
| Y264H | rs1260764171 | TAC > CAC | Deleterious | -4.71 | probably damaging | 1 | Disease | 0.815 |
| Y379C | rs1378241611 | TAT > TGT | Deleterious | -7.39 | probably damaging | 1 | Disease | 0.821 |
| R230C | rs771876073 | CGT > TGT | Deleterious | -6.42 | probably damaging | 1 | Disease | 0.828 |
| R230S | rs771876073 | CGT > AGT | Deleterious | -4.1 | probably damaging | 1 | Disease | 0.803 |
| R230L | rs201985154 | CGT > CTT | Deleterious | -5.53 | probably damaging | 1 | Disease | 0.881 |
| R365W | rs769857663 | CGG > TGG | Deleterious | -5.35 | probably damaging | 1 | Disease | 0.795 |
| R263S | rs761608312 | CGC > AGC | Deleterious | -4 | probably damaging | 1 | Disease | 0.875 |
| R204W | rs747846898 | CGG > TGG | Deleterious | -5.31 | probably damaging | 1 | Disease | 0.787 |
| S184F | rs754390998 | TCC > TTC | Deleterious | -4.21 | probably damaging | 1 | Disease | 0.54 |
| E201G | rs1273601119 | GAG>GGG | Deleterious | -4.53 | probably damaging | 1 | Disease | 0.706 |
| R273P | rs778068396 | CGG > CCG | Deleterious | -3.83 | probably damaging | 1 | Disease | 0.908 |
| R227W | rs1222300931 | CGG > TGG | Deleterious | -5.8 | probably damaging | 1 | Disease | 0.841 |
| L134P | rs367710814 | CTC > CCC | Deleterious | -4.83 | probably damaging | 1 | Disease | 0.911 |
| L304V | rs769367192 | CTC > GTC | Deleterious | -2.55 | probably damaging | 1 | Disease | 0.74 |
| R328C | rs1310479893 | CGT > TGT | Deleterious | -7.7 | probably damaging | 1 | Disease | 0.953 |
| L191W | rs994376888 | TTG > TGG | Deleterious | -5.34 | probably damaging | 1 | Disease | 0.857 |
| Q255L | rs763144708 | CAG > CTG | Deleterious | -4.93 | probably damaging | 0.998 | Disease | 0.879 |
| Y126C | rs1283314818 | TAC > TGC | Deleterious | -4.91 | probably damaging | 1 | Disease | 0.9 |
| D285E | rs1056097466 | GAC > GAA | Deleterious | -3.74 | probably damaging | 1 | Disease | 0.828 |
| A316G | rs748335405 | GCC > GGC | Deleterious | -3.71 | probably damaging | 1 | Disease | 0.664 |
| R263C | rs761608312 | CGC > TGC | Deleterious | -6.08 | probably damaging | 0.996 | Disease | 0.879 |
| G250S | rs751767798 | GGC > AGC | Deleterious | -4.37 | probably damaging | 1 | Disease | 0.713 |
| D331N | rs1373347535 | GAT > AAT | Deleterious | -4.68 | probably damaging | 1 | Disease | 0.903 |
| R263G | rs761608312 | CGC > GGC | Deleterious | -5.09 | probably damaging | 1 | Disease | 0.87 |
| E209K | rs370297398 | GAG > AAG | Deleterious | -3.42 | probably damaging | 1 | Disease | 0.742 |
| R327Q | rs1246408404 | CGA > CAA | Deleterious | -3.84 | probably damaging | 0.975 | Disease | 0.919 |
| R269G | rs748929277 | AGG >GGG | Deleterious | -5.43 | probably damaging | 0.999 | Disease | 0.869 |
| R359W | rs200104639 | CGG > TGG | Deleterious | -5.12 | probably damaging | 1 | Disease | 0.793 |
| R371W | rs1186523174 | CGG > TGG | Deleterious | -5.48 | probably damaging | 1 | Disease | 0.757 |
| L352P | rs984036888 | CTA > CCA | Deleterious | -3.97 | probably damaging | 1 | Disease | 0.92 |

Supplementary Table 4: Predictions of SNAP2, PredictSNP, Panther, & PMut for the 39 High Risk nsSNPs of DAXX protein.

| AA Variant | SNAP2 | | PredictSNP | | Panther | PMut | |
| --- | --- | --- | --- | --- | --- | --- | --- |
|  | Prediction | Score | Prediction | Accuracy | Prediction | Disease Prediction | Score |
| C74F | effect | 74 | DELETERIOUS | 0.86908365 | probably damaging | TRUE | 0.8585 |
| D219V | effect | 53 | DELETERIOUS | 0.7556615 | probably damaging | TRUE | 0.8585 |
| S220C | effect | 45 | DELETERIOUS | 0.71871275 | probably damaging | TRUE | 0.8555 |
| S102L | effect | 81 | DELETERIOUS | 0.86908365 | probably damaging | TRUE | 0.8464 |
| R251C | effect | 64 | DELETERIOUS | 0.86908365 | probably damaging | TRUE | 0.8331 |
| R328H | effect | 69 | DELETERIOUS | 0.86908365 | probably damaging | TRUE | 0.8009 |
| R251H | effect | 73 | DELETERIOUS | 0.86908365 | probably damaging | TRUE | 0.7993 |
| P284S | effect | 71 | DELETERIOUS | 0.7556615 | probably damaging | TRUE | 0.7993 |
| E209Q | effect | 17 | DELETERIOUS | 0.71871275 | possibly damaging | TRUE | 0.781 |
| Y264H | effect | 88 | DELETERIOUS | 0.71871275 | probably damaging | TRUE | 0.7476 |
| Y379C | effect | 37 | DELETERIOUS | 0.7556615 | probably damaging | TRUE | 0.6921 |
| R230C | effect | 63 | DELETERIOUS | 0.7556615 | possibly damaging | TRUE | 0.6739 |
| R230S | effect | 70 | DELETERIOUS | 0.7556615 | possibly damaging | TRUE | 0.6739 |
| R230L | effect | 70 | DELETERIOUS | 0.7556615 | possibly damaging | TRUE | 0.6739 |
| R365W | effect | 46 | DELETERIOUS | 0.86908365 | probably damaging | TRUE | 0.6739 |
| R263S | effect | 60 | DELETERIOUS | 0.50595948 | probably damaging | TRUE | 0.664 |
| R204W | effect | 42 | DELETERIOUS | 0.86908365 | possibly damaging | TRUE | 0.6613 |
| S184F | effect | 16 | DELETERIOUS | 0.71871275 | possibly damaging | TRUE | 0.6603 |
| E201G | effect | 70 | DELETERIOUS | 0.65494636 | possibly damaging | TRUE | 0.654 |
| R273P | effect | 66 | DELETERIOUS | 0.86908365 | possibly damaging | TRUE | 0.6495 |
| R227W | effect | 63 | DELETERIOUS | 0.60697259 | possibly damaging | TRUE | 0.6372 |
| L134P | effect | 83 | DELETERIOUS | 0.86908365 | probably damaging | TRUE | 0.6314 |
| L304V | effect | 45 | DELETERIOUS | 0.65494636 | probably damaging | TRUE | 0.6286 |
| R328C | effect | 64 | DELETERIOUS | 0.86908365 | probably damaging | TRUE | 0.6228 |
| L191W | effect | 66 | DELETERIOUS | 0.7556615 | probably damaging | TRUE | 0.6203 |
| Q255L | effect | 32 | DELETERIOUS | 0.60548272 | possibly damaging | TRUE | 0.6135 |
| Y126C | effect | 71 | DELETERIOUS | 0.7556615 | probably damaging | TRUE | 0.6131 |
| D285E | effect | 60 | DELETERIOUS | 0.86908365 | probably damaging | TRUE | 0.599 |
| A316G | effect | 48 | DELETERIOUS | 0.65494636 | probably damaging | TRUE | 0.5855 |
| R263C | effect | 49 | DELETERIOUS | 0.71871275 | probably damaging | TRUE | 0.5793 |
| G250S | effect | 59 | DELETERIOUS | 0.60548272 | probably damaging | TRUE | 0.5749 |
| D331N | effect | 74 | DELETERIOUS | 0.71871275 | probably damaging | TRUE | 0.5707 |
| R263G | effect | 62 | DELETERIOUS | 0.50595948 | probably damaging | TRUE | 0.5524 |
| E209K | effect | 48 | DELETERIOUS | 0.50595948 | possibly damaging | TRUE | 0.5483 |
| R327Q | effect | 59 | DELETERIOUS | 0.54946365 | probably damaging | TRUE | 0.5399 |
| R269G | effect | 70 | DELETERIOUS | 0.7556615 | possibly damaging | TRUE | 0.5194 |
| R359W | effect | 30 | DELETERIOUS | 0.62127533 | possibly damaging | TRUE | 0.5035 |
| R371W | effect | 46 | DELETERIOUS | 0.7556615 | possibly damaging | TRUE | 0.5035 |
| L352P | effect | 23 | DELETERIOUS | 0.7556615 | probably damaging | TRUE | 0.5006 |

Supplementary Table 5: Predictions of PROVEAN, PolyPhen-2 & SNPs&GO for the 15 High Risk nsSNPs of ATRX protein.

| AA Variant | rs ID | Codon | PROVEAN | | PolyPhen-2 | | SNPs&GO | |
| --- | --- | --- | --- | --- | --- | --- | --- | --- |
|  |  |  | **Prediction** | **Score** | **Prediction** | **Probability2** | **Prediction** | **Probability** |
| C200F | rs886041700 | TGC > TTC | Deleterious | -5.87 | probably damaging | 0.999 | Disease | 0.955 |
| C220Y | rs122445111 | TGT > TAT | Deleterious | -5.83 | probably damaging | 1 | Disease | 0.927 |
| C223Y | rs1557142844 | TGT > TAT | Deleterious | -6.13 | probably damaging | 0.999 | Disease | 0.955 |
| C231Y | rs1353833841 | TGT > TAT | Deleterious | -5.58 | probably damaging | 0.998 | Disease | 0.965 |
| C243W | rs1057524153 | TGC > TGG | Deleterious | -6.13 | probably damaging | 1 | Disease | 0.959 |
| C280R | rs797045408 | TGT > CGT | Deleterious | -6.69 | probably damaging | 0.999 | Disease | 0.913 |
| D115V | rs1557149892 | GAT > GTT | Deleterious | -2.95 | probably damaging | 0.999 | Disease | 0.576 |
| G337C | rs977747361 | GGC > TGC | Deleterious | -4.62 | probably damaging | 0.986 | Disease | 0.671 |
| N237H | rs781810798 | AAT > CAT | Deleterious | -2.65 | probably damaging | 0.999 | Disease | 0.774 |
| N367Y | rs1557142343 | AAC > TAC | Deleterious | -4.04 | probably damaging | 0.999 | Disease | 0.865 |
| P190L | rs1057518708 | CCT > CTT | Deleterious | -5.52 | probably damaging | 1 | Disease | 0.832 |
| R246C | rs122445105 | CGC > TGC | Deleterious | -4.46 | probably damaging | 1 | Disease | 0.921 |
| R654G | rs1557140492 | CGA > GGA | Deleterious | -3.52 | probably damaging | 0.997 | Disease | 0.564 |
| V178D | rs1060499759 | GTC > GAC | Deleterious | -3.78 | probably damaging | 1 | Disease | 0.852 |
| V277G | rs797044793 | GTC > GGC | Deleterious | -3.63 | probably damaging | 1 | Disease | 0.606 |

Supplementary Table 6: Predictions of SNAP2, PredictSNP, Panther, & PMut for the 15 High Risk nsSNPs of ATRX protein.

| AA Variant | SNAP2 | | PredictSNP | | Panther | PMut | |
| --- | --- | --- | --- | --- | --- | --- | --- |
|  | **Prediction** | **Score** | **Prediction** | **Accuracy** | **Prediction** | **Disease Prediction** | **Score** |
| C200F | effect | 44 | DELETERIOUS | 0.869084 | probably damaging | TRUE | 0.8528 |
| C220Y | effect | 49 | DELETERIOUS | 0.869084 | probably damaging | TRUE | 0.7448 |
| C223Y | effect | 59 | DELETERIOUS | 0.755662 | probably damaging | TRUE | 0.8635 |
| C231Y | effect | 48 | DELETERIOUS | 0.869084 | probably damaging | TRUE | 0.8581 |
| C243W | effect | 66 | DELETERIOUS | 0.869084 | probably damaging | TRUE | 0.8704 |
| C280R | effect | 53 | DELETERIOUS | 0.869084 | probably damaging | TRUE | 0.8635 |
| D115V | effect | 51 | DELETERIOUS | 0.654946 | possibly damaging | TRUE | 0.5315 |
| G337C | effect | 53 | DELETERIOUS | 0.654946 | probably damaging | TRUE | 0.8399 |
| N237H | effect | 16 | DELETERIOUS | 0.718713 | probably damaging | TRUE | 0.8514 |
| N367Y | effect | 43 | DELETERIOUS | 0.755662 | probably damaging | TRUE | 0.7479 |
| P190L | effect | 12 | DELETERIOUS | 0.755662 | probably damaging | TRUE | 0.8528 |
| R246C | effect | 58 | DELETERIOUS | 0.869084 | probably damaging | TRUE | 0.8704 |
| R654G | effect | 36 | DELETERIOUS | 0.505959 | probably damaging | TRUE | 0.5605 |
| V178D | effect | 40 | DELETERIOUS | 0.718713 | probably damaging | TRUE | 0.8581 |
| V277G | effect | 45 | DELETERIOUS | 0.755662 | probably damaging | TRUE | 0.8701 |

Supplementary Table 7: Molecular Effects of the High Risk nsSNPs on their respective proteins, SMARCAL1, DAXX and ATRX.

| **SMARCAL1** | | **DAXX** | | **ATRX** | |
| --- | --- | --- | --- | --- | --- |
| **AA Variant** | **Molecular mechanisms with P-values <= 0.05** | **AA Variant** | **Molecular mechanisms with P-values <= 0.05** | **AA Variant** | **Molecular mechanisms with P-values <= 0.05** |
|  |  |  |  |  |  |
| **R23C** | Altered Coiled coil  Altered Disordered interface  Loss of Acetylation at K27  Loss of SUMOylation at K27  Loss of Methylation at K19 | **C74F** | Altered Disordered interface  Altered Coiled coil | **C200F** | Altered Metal binding  Altered Disordered interface  Altered Ordered interface  Altered Transmembrane protein |
|  |  | **D219V** | Loss of Intrinsic disorder  Altered Disordered interface  Loss of B-factor  Loss of Phosphorylation at Y222  Altered Coiled coil  Loss of Sulfation at Y222 |  |  |
|  |  |  |  | **C220Y** | Altered Metal binding  Altered Ordered interface  Gain of Disulfide linkage at C223  Altered Transmembrane protein  Loss of Pyrrolidone carboxylic acid at Q219 |
| **G239R** | Altered Disordered interface  Gain of GPI-anchor amidation at N235 |  |  |  |  |
| **R247C** | Altered Metal binding  Altered Transmembrane protein |  |  |  |  |
|  |  | **D285E** | Altered Disordered interface  Loss of Phosphorylation at Y286  Loss of Proteolytic cleavage at D285  Loss of Sulfation at Y286 |  |  |
| **R247P** | Altered Transmembrane protein |  |  | **C223Y** | Altered Metal binding  Altered Ordered interface  Gain of Strand  Altered Transmembrane protein  Gain of Disulfide linkage at C220  Loss of Pyrrolidone carboxylic acid at Q219 |
| **Y254S** | Altered Ordered interface |  |  |  |  |
| **F343S** | Altered Transmembrane protein  Altered Stability |  |  |  |  |
|  |  | **D331N** | Loss of Sulfation at Y334 |  |  |
| **G443R** | Gain of Helix | **E201G** | - |  |  |
| **G443A** |  | **G250S** | - |  |  |
| **A457T** | Gain of Relative solvent accessibility  Altered Ordered interface  Gain of Strand  Altered Metal binding  Gain of Allosteric site at D458  Gain of Catalytic site at G461 | **L134P** | Gain of Intrinsic disorder  Gain of Helix  Altered Stability | **C231Y** | Altered Metal binding  Gain of Strand  Loss of Helix  Altered Transmembrane protein  Gain of Disulfide linkage at C232  Gain of Sulfation at C231 |
|  |  | **L191W** | Altered Coiled coil  Loss of Intrinsic disorder  Altered Disordered interface |  |  |
| **D458E** | Altered Metal binding  Loss of Allosteric site at M460  Gain of Strand  Gain of Catalytic site at R453 | **P284S** | Altered Disordered interface  Gain of Relative solvent accessibility  Gain of Phosphorylation at Y286  Altered DNA binding  Gain of Proteolytic cleavage at D285  Loss of Sulfation at Y286 | **C243W** | Altered Disordered interface  Altered Metal binding  Altered Transmembrane protein  Gain of Disulfide linkage at C240  Altered DNA binding |
| **G463E** | Loss of Catalytic site at K464  Gain of Allosteric site at D458  Altered Ordered interface  Altered Metal binding  Loss of Methylation at K464 |  |  |  |  |
|  |  |  |  | **C280R** | Altered Coiled coil |
|  |  | **R227W** | Altered Disordered interface  Loss of Phosphorylation at Y222  Loss of Helix  Gain of Sulfation at Y222 | **D115V** | Loss of Phosphorylation at S112  Altered Disordered interface  Loss of B-factor |
| **K464R** | Altered Ordered interface  Loss of Catalytic site at K464  Loss of Allosteric site at M460  Altered Metal binding  Loss of Methylation at K464 |  |  | **G337C** | Loss of Proteolytic cleavage at Y341 |
|  |  | **R230C** | - | **N237H** | Altered Metal binding  Altered Transmembrane protein  Loss of Disulfide linkage at C232 |
|  |  | **R230L** | - |  |  |
|  |  | **R230S** | - |  |  |
|  |  | **R251C** | Altered DNA binding  Loss of Proteolytic cleavage at R251 | **N367Y** | Altered Disordered interface  Altered Coiled coil  Altered Ordered interface  Altered Transmembrane protein  Loss of Acetylation at K372  Loss of N-linked glycosylation at N367 |
| **T465I** | Loss of Catalytic site at K464  Loss of Allosteric site at M460  Altered Metal binding  Loss of Methylation at K464 |  |  |  |  |
|  |  | **R251H** | Gain of Strand  Altered DNA binding  Loss of Proteolytic cleavage at R251 |  |  |
| **R476W** | - | **R269G** | Altered Disordered interface  Altered DNA binding  Altered Stability |  |  |
| **P480L** | Altered Ordered interface  Gain of Strand  Loss of Loop |  |  | **P190L** | Loss of Strand  Altered Ordered interface  Gain of Allosteric site at H189  Altered Metal binding  Altered DNA binding |
|  |  | **R273P** | Altered Disordered interface  Altered DNA binding  Loss of Helix  Loss of Relative solvent accessibility  Loss of Acetylation at K277  Gain of Ubiquitylation at K277  Altered Stability |  |  |
| **S487F** | Altered DNA binding |  |  |  |  |
| **R499W** | Gain of Pyrrolidone carboxylic acid at Q495 |  |  |  |  |
|  |  |  |  | **R246C** | Altered Metal binding  Altered Transmembrane protein  Altered DNA binding  Gain of Amidation at L248 |
| **P502Q** | Gain of Strand |  |  |  |  |
| **I547T** | Loss of Catalytic site at E550  Gain of Allosteric site at E550  Altered Metal binding  Altered Stability |  |  |  |  |
|  |  | **R327Q** | --- | **R654G** | Altered Disordered interface |
|  |  | **R328C** | Loss of Helix  Altered Disordered interface | **V178D** | Gain of Catalytic site at C174  Altered Transmembrane protein |
| **I548N** | Gain of Allosteric site at D549  Altered Ordered interface  Loss of Strand  Altered Metal binding  Loss of Catalytic site at D549  Altered Stability  Gain of Acetylation at K544 |  |  |  |  |
|  |  | **R328H** | Loss of Helix  Altered Metal binding | **V277G** | Altered Stability |
|  |  |  |  |  |  |
|  |  | **S102L** | Gain of Helix |  |  |
|  |  | **Y126C** | Altered Ordered interface  Altered Metal binding  Altered Coiled coil  Altered Transmembrane protein  Altered Stability |  |  |
| **L569P** | Altered Disordered interface  Loss of Acetylation at K570 |  |  |  |  |
| **I576T** | Altered Stability  Altered Ordered interface  Gain of Catalytic site at S579  Altered DNA binding  Gain of Allosteric site at T581  Altered Disordered interface  Altered Metal binding | **Y264H** | Altered Disordered interface  Altered DNA binding |  |  |
|  |  | **S220C** | Loss of Intrinsic disorder  Altered Disordered interface  Loss of B-factor  Loss of Phosphorylation at Y222  Loss of Sulfation at Y222 |  |  |
| **L578S** | Altered Ordered interface  Gain of Catalytic site at S579  Altered DNA binding  Loss of Allosteric site at T581  Altered Disordered interface  Altered Metal binding  Altered Stability | **E209Q** | - |  |  |
|  |  | **Y379C** | Altered Disordered interface  Loss of Phosphorylation at Y379  Altered Coiled coil  Loss of Acetylation at K378  Loss of Ubiquitylation at K378 |  |  |
|  |  | **R365W** | - |  |  |
| **T581S** | Gain of Catalytic site at S579  Altered DNA binding  Loss of Allosteric site at T581  Altered Metal binding | **R263S** | Gain of Strand  Altered DNA binding |  |  |
|  |  | **R204W** | - |  |  |
|  |  | **S184F** | - |  |  |
| **P582A** | Altered Ordered interface  Gain of Helix  Loss of Catalytic site at S579  Altered DNA binding  Loss of Allosteric site at T581  Altered Metal binding | **L304V** | - |  |  |
|  |  | **Q255L** | - |  |  |
|  |  | **A316G** | - |  |  |
|  |  | **R263C** | Gain of Strand  Altered DNA binding  Gain of Strand  Altered DNA binding |  |  |
|  |  | **R263G** |  |  |  |
| **P582S** | Altered Ordered interface  Gain of Catalytic site at S579  Altered DNA binding  Altered Metal binding  Gain of Allosteric site at T581 |  |  |  |  |
|  |  | **E209K** | - |  |  |
|  |  | **R359W** | - |  |  |
|  |  | **R371W** | - |  |  |
|  |  | **L352P** | Gain of Intrinsic disorder  Gain of B-factor  Gain of Loop  Altered Metal binding  Altered Coiled coil  Altered Stability |  |  |
| **R586W** | Loss of Catalytic site at T581  Loss of Allosteric site at T581  Altered DNA binding |  |  |  |  |
| **C613Y** | Altered Transmembrane protein  Loss of Disulfide linkage at C613 |  |  |  |  |
| **G621R** | Altered Transmembrane protein  Gain of Proteolytic cleavage at D623 |  |  |  |  |
| **L636P** | Altered Stability |  |  |  |  |
| **R644Q** | Gain of Strand  Altered DNA binding |  |  |  |  |
| **R644W** | Gain of Strand  Altered DNA binding |  |  |  |  |
| **R645H** | Gain of Strand  Altered DNA binding |  |  |  |  |
| **R645C** | Loss of Allosteric site at R645  Altered DNA binding |  |  |  |  |
| **K647E** | Altered DNA binding |  |  |  |  |
| **R659C** | Altered Disordered interface |  |  |  |  |

Supplementary Table 8: Prediction of PTMs at the High Risk nsSNP residue positions of DAXX, SMARCAL1 and ATRX protein.

| **DAXX** | | **SMARCAL1** | | **ATRX** | |
| --- | --- | --- | --- | --- | --- |
| **Residues** | PTMs | **Residues** | PTMs | **Residues** | PTMs |
| **D219** | Proteolytic_cleavage | **C613** | Disulfide_linkage | **C200** | Disulfide_linkage |
| **D285** | Proteolytic_cleavage | **D458** | Proteolytic_cleavage | **C220** | Disulfide_linkage |
| **D331** | Proteolytic_cleavage | **K464** | Ubiquitination | **C223** | Disulfide_linkage |
| **E201** | Carboxylation | **K464** | Proteolytic_cleavage | **C231** | Palmitoylation |
| **L352** | Proteolytic_cleavage | **K647** | Proteolytic_cleavage | **C231** | Disulfide_linkage |
| **P284** | Proteolytic_cleavage | **K647** | Methylation | **C243** | Disulfide_linkage |
| **Q255** | Proteolytic_cleavage | **L578** | Proteolytic_cleavage | **C243** | Palmitoylation |
| **R204** | Proteolytic_cleavage | **R23** | Proteolytic_cleavage | **D115** | Proteolytic_cleavage |
| **R227** | Proteolytic_cleavage | **R23** | ADP-ribosylation | **G337** | Proteolytic_cleavage |
| **R230** | ADP-ribosylation | **R247** | Proteolytic_cleavage | **N367** | N-linked_glycosylation |
| **R251** | Proteolytic_cleavage | **R247** | Methylation | **N367** | GPI_anchor_amidation |
| **R263** | Proteolytic_cleavage | **R499** | Proteolytic_cleavage | **P190** | Proteolytic_cleavage |
| **R269** | Methylation | **R586** | ADP-ribosylation | **R246** | Proteolytic_cleavage |
| **R273** | Proteolytic_cleavage | **R644** | ADP-ribosylation | **R654** | ADP-ribosylation |
| **R359** | Proteolytic_cleavage | **R644** | Proteolytic_cleavage | **R654** | Proteolytic_cleavage |
| **R359** | ADP-ribosylation | **R645** | Proteolytic_cleavage |  |  |
| **R365** | Proteolytic_cleavage | **R659** | ADP-ribosylation |  |  |
| **R371** | ADP-ribosylation | **R659** | Proteolytic_cleavage |  |  |
| **R371** | Methylation | **T581** | Phosphorylation |  |  |
| **R371** | Proteolytic_cleavage | **T581** | O-linked_glycosylation |  |  |
| **S184** | O-linked_glycosylation |  |  |  |  |
| **S220** | Phosphorylation |  |  |  |  |
| **S220** | Proteolytic_cleavage |  |  |  |  |
| **Y264** | Phosphorylation |  |  |  |  |
| **Y379** | Phosphorylation |  |  |  |  |
| **Y379** | Proteolytic_cleavage |  |  |  |  |

Supplementary Table 9: Prediction of Protein Stability of SMARCAL1 protein by MuPro and I-Mutant and Conservation status by Consurf. [e=exposed, f=functional, b=buried, s=structural residue; 9 is the highest conservation score].

| **AA Variant** | **MuPro** | | **I-Mutant** | | **Conservation Status** |
| --- | --- | --- | --- | --- | --- |
|  | Prediction | DDG Value (Kcal/mol) | Prediction | DDG Value (Kcal/mol) |  |
| **I547T** | Decrease | -2.052 | Decrease | -2.14 | 9 (b, s) |
| **A457T** | Decrease | -1.786 | Decrease | -0.47 | 9 (b,s) |
| **D458E** | Decrease | -1.649 | Increase | 0.03 | 9 (e,f) |
| **L569P** | Decrease | -1.62 | Decrease | -1.54 | 9 (b,s) |
| **L578S** | Decrease | -1.58 | Decrease | -2.42 | 9 (b,s) |
| **Y254S** | Decrease | -1.548 | Decrease | -1.55 | 9 (b,s) |
| **F343S** | Decrease | -1.409 | Decrease | -1.85 | 9 (b) |
| **R645H** | Decrease | -1.32 | Decrease | -1.14 | 9 (e,f) |
| **C613Y** | Decrease | -1.241 | Decrease | 0 | 9 (b,s) |
| **G443A** | Decrease | -1.226 | Decrease | -0.81 | 9 (e,f) |
| **G443R** | Decrease | -1.16 | Decrease | -0.59 | 9 (e,f) |
| **R247P** | Decrease | -1.147 | Decrease | -0.95 | 9 (e,f) |
| **R645C** | Decrease | -1.13 | Decrease | -0.74 | 9 (e,f) |
| **R23C** | Decrease | -1.097 | Decrease | -0.43 | 9 (e,f) |
| **P582A** | Decrease | -1.08 | Decrease | -1.6 | 9 (e,f) |
| **G239R** | Decrease | -1.044 | Decrease | -0.72 | 9 (e,f) |
| **R644Q** | Decrease | -0.999 | Decrease | -0.6 | 9 (e,f) |
| **P502Q** | Decrease | -0.955 | Decrease | -1.45 | 9 (e,f) |
| **R644W** | Decrease | -0.95 | Decrease | -0.22 | 9 (e,f) |
| **G621R** | Decrease | -0.938 | Decrease | -0.5 | 9 (b,s) |
| **T581S** | Decrease | -0.889 | Decrease | -0.77 | 9 (b,s) |
| **P582S** | Decrease | -0.817 | Decrease | -1.88 | 9 (e,f) |
| **R586W** | Decrease | -0.756 | Decrease | -0.23 | 9 (e,f) |
| **R247C** | Decrease | -0.724 | Decrease | -1.25 | 9 (e,f) |
| **K647E** | Decrease | -0.596 | Decrease | -0.03 | 9 (e,f) |
| **G463E** | Decrease | -0.563 | Decrease | -0.42 | 9 (e,f) |
| **R659C** | Decrease | -0.374 | Decrease | -0.96 | 9 (e,f) |
| **P480L** | Decrease | -0.196 | Decrease | -0.71 | 9 (e,f) |
| **K464R** | Increase | 0.102 | Decrease | -0.09 | 9 (e,f) |
| **T465I** | Increase | 0.172 | Decrease | -0.19 | 9 (b,s) |
| **S487F** | Increase | 0.387 | Increase | 0.44 | 9 (e,f) |
| **I576T** | Decrease | -2.33 | Decrease | -2.1 | 8 (b) |
| **L636P** | Decrease | -2.19 | Decrease | -1.36 | 8 (b) |
| **I548N** | Decrease | -1.935 | Decrease | -2 | 7 (b) |
| **R476W** | Decrease | -0.888 | Decrease | -0.27 | 6 (e) |
| **R499W** | Decrease | -0.809 | Decrease | -0.21 | 6 (e) |

Supplementary Table 10: Prediction of Protein Stability of DAXX protein by MuPro and I-Mutant and Conservation status by Consurf.

| **AA Variant** | **MuPro** | | **I-Mutant** | | **Conservation Status** |
| --- | --- | --- | --- | --- | --- |
|  | Prediction | DDG Value (Kcal/mol) | Prediction | DDG Value (Kcal/mol) |  |
| **C74F** | Decrease | -1.23 | Increase | 0.37 | 9 (b, s) |
| **D219V** | Decrease | -0.11 | Increase | 0.13 | 9 (e,f) |
| **D285E** | Decrease | -0.95 | Decrease | -0.39 | 9 (e,f) |
| **D331N** | Decrease | -1.73 | Decrease | -0.76 | 9 (e,f) |
| **E201G** | Decrease | -1.52 | Decrease | -0.78 | 9 (e,f) |
| **G250S** | Decrease | -1.33 | Decrease | -1.35 | 9 (b,s) |
| **L134P** | Decrease | -1.89 | Decrease | -1.38 | 8 (b) |
| **L191W** | Decrease | -1.49 | Decrease | -1.21 | 9 (b,s) |
| **P284S** | Decrease | -1.09 | Decrease | -1.98 | 9 (e,f) |
| **R227W** | Decrease | -0.86 | Decrease | -0.31 | 8 (e,f) |
| **R230C** | Decrease | -1.09 | Decrease | -0.76 | 9 (e,f) |
| **R230L** | Decrease | -0.44 | Decrease | -0.31 | 9 (e,f) |
| **R230S** | Decrease | -1.29 | Decrease | -1 | 9 (e,f) |
| **R251C** | Decrease | -0.83 | Decrease | -1.2 | 9 (e,f) |
| **R251H** | Decrease | -1.31 | Decrease | -1.55 | 9 (e,f) |
| **R269G** | Decrease | -1.81 | Decrease | -1.69 | 7 (e) |
| **R273P** | Decrease | -1.55 | Decrease | -1.01 | 8 (e,f) |
| **R327Q** | Decrease | -1.01 | Decrease | -1.19 | 9 (e,f) |
| **R328C** | Decrease | -1.65 | Decrease | -1.2 | 9 (e,f) |
| **R328H** | Decrease | -1.96 | Decrease | -1.49 | 9 (e,f) |
| **S102L** | Increase | 0.38 | Increase | 0.13 | 9 (b,s) |
| **Y126C** | Decrease | -1.32 | Decrease | -1.32 | 7 (b) |
| **Y264H** | Decrease | -1.36 | Decrease | -1.16 | 9 (e,f) |
| **S220C** | Decrease | -0.14 | Decrease | -0.37 | 9 (e,f) |
| **E209Q** | Decrease | -0.514 | Decrease | -0.45 | 8 (e,f) |
| **Y379C** | Decrease | -0.647 | Decrease | -0.87 | 7 (b) |
| **R365W** | Decrease | -0.931 | Decrease | -0.5 | 9 (e,f) |
| **R263S** | Decrease | -1.143 | Decrease | -1.3 | 9 (e,f) |
| **R204W** | Decrease | -1.12 | Decrease | -0.26 | 9 (e,f) |
| **S184F** | Decrease | -0.089 | Increase | 0.5 | 9 (e,f) |
| **L304V** | Decrease | -0.667 | Decrease | -1.22 | 9 (b,s) |
| **Q255L** | Increase | 0.251 | Decrease | -0.05 | 8 (e,f) |
| **A316G** | Decrease | -1.902 | Decrease | -0.91 | 9 (b,s) |
| **R263C** | Decrease | -0.823 | Decrease | -0.96 | 9 (e,f) |
| **R263G** | Decrease | -1.257 | Decrease | -1.44 | 9 (e,f) |
| **E209K** | Decrease | -0.913 | Decrease | -0.71 | 8 (e,f) |
| **R359W** | Decrease | -0.927 | Decrease | -0.44 | 7 (e) |
| **R371W** | Decrease | -0.726 | Decrease | -0.66 | 6 (e) |
| **L352P** | Decrease | -1.86 | Decrease | -1.8 | 8 (b) |

Supplementary Table 11: Prediction of Protein Stability of ATRX protein by MuPro and I-Mutant and Conservation status by Consurf.

| **AA Variant** | **Mupro** | | **I-Mutant** | | **Conservation Status** |
| --- | --- | --- | --- | --- | --- |
|  | Prediction | DDG Value (Kcal/mol) | Prediction | DDG Value (Kcal/mol) |  |
| **C200F** | Decrease | -0.959 | Decrease | -0.21 | 9(b,s) |
| **C220Y** | Decrease | -0.396 | Decrease | -0.27 | 9 (b,s) |
| **C223Y** | Decrease | -0.884 | Increase | -0.07 | 9 (e,f) |
| **C243W** | Decrease | -0.557 | Decrease | -0.21 | 9 (b,s) |
| **C280R** | Decrease | -1.75 | Decrease | -0.42 | 9 (b,s) |
| **R246C** | Decrease | -0.637 | Decrease | -0.97 | 9 (e,f) |
| **R654G** | Decrease | -1.855 | Decrease | -1.45 | 9 (e,f) |
| **V178D** | Decrease | -0.767 | Decrease | -1.07 | 9 (b,s) |
| **V277G** | Decrease | -2.665 | Decrease | -2.51 | 9 (b,s) |
| **N237H** | Decrease | -0.657 | Decrease | -0.7 | 8 (e,f) |
| **D115V** | Decrease | -0.668 | Decrease | -0.21 | 7 (e) |
| **P190L** | Increase | 0.28 | Decrease | -0.74 | 7 (e) |
| **C231Y** | Decrease | -0.738 | Increase | -0.07 | 6 (b) |
| **N367Y** | Decrease | -0.77 | Increase | 0.31 | 6 (e) |
| **G337C** | Decrease | -0.508 | Decrease | -1.04 | 4 (e) |
